# Meta-analysis with zero-event studies: a comparative study with application to COVID-19 data

**DOI:** 10.1186/s40779-021-00331-6

**Published:** 2021-07-03

**Authors:** Jia-Jin Wei, En-Xuan Lin, Jian-Dong Shi, Ke Yang, Zong-Liang Hu, Xian-Tao Zeng, Tie-Jun Tong

**Affiliations:** 1 Department of Mathematics, Hong Kong Baptist University, Hong Kong, China; 2 Shenzhen Research Institute of Big Data, Shenzhen, China; 3 College of Mathematics and Statistics, Shenzhen University, Shenzhen, China; 4 Center for Evidence-Based and Translational Medicine, Zhongnan Hospital of Wuhan University, Wuhan, China

**Keywords:** Continuity correction, Coronavirus disease 2019 data, Meta-analysis, Relative risk, Zero-event studies

## Abstract

**Background:**

Meta-analysis is a statistical method to synthesize evidence from a number of independent studies, including those from clinical studies with binary outcomes. In practice, when there are zero events in one or both groups, it may cause statistical problems in the subsequent analysis.

**Methods:**

In this paper, by considering the relative risk as the effect size, we conduct a comparative study that consists of four continuity correction methods and another state-of-the-art method without the continuity correction, namely the generalized linear mixed models (GLMMs). To further advance the literature, we also introduce a new method of the continuity correction for estimating the relative risk.

**Results:**

From the simulation studies, the new method performs well in terms of mean squared error when there are few studies. In contrast, the generalized linear mixed model performs the best when the number of studies is large. In addition, by reanalyzing recent coronavirus disease 2019 (COVID-19) data, it is evident that the double-zero-event studies impact the estimate of the mean effect size.

**Conclusions:**

We recommend the new method to handle the zero-event studies when there are few studies in a meta-analysis, or instead use the GLMM when the number of studies is large. The double-zero-event studies may be informative, and so we suggest not excluding them.

**Supplementary Information:**

The online version contains supplementary material available at (10.1186/s40779-021-00331-6).

## Background

Meta-analysis is a statistical method to synthesize evidence from a number of independent studies that addressed the same scientific questions [1, 2]. In clinical studies, experimental data are commonly composed of binary outcomes, and consequently, meta-analyses of binary data have attracted increasing attention in evidence-based medicine [3, 4]. For each study, an effect size is reported to quantify the treatment effect by comparing the event probabilities between the treatment group and the control group, including the odds ratio (OR), the relative risk (RR), and the risk difference (RD). In meta-analysis, when the study-specific effect size is estimated based on a two-by-two contingency table, the zero-event problem in one or both groups frequently occurs, which may cause an unexpected calculation complication in the statistical inference of the effect size. If the study involves a zero event in one group, we refer to it as a *single-zero-event study*; and if the study involves zero events in both groups, we refer to it as a *double-zero-event study* [5]. Vandermeer et al. [6] and Kuss [7] applied random sampling techniques and found that 30% of meta-analyses from the 500 sampled Cochrane reviews included one or more single-zero-event studies, while 34% of the reviews involved at least one meta-analysis with a double-zero-event study.

As a recent example, Chu et al. [8] conducted several meta-analyses to evaluate the effectiveness of physical distancing, face masks, and eye protection on the spread of three coronaviruses, which caused severe acute respiratory syndrome (SARS), Middle East respiratory syndrome (MERS) or coronavirus disease 2019, also known as COVID-19 [9, 10]. Specifically, they considered RR as the effect size and applied the random-effects model to pool the observed effect sizes with an inverse-variance weight assigned to each study [11, 12]. As a result, for their meta-analysis on physical distancing, they concluded that the risk of infection will be significantly decreased with a further physical distance. We note, however, that there are 8 single-zero-event studies and 7 double-zero-event studies among a total of 32 studies. In particular for the 7 studies on COVID-19 data, 4 of them are single-zero-event studies and 2 of them are double-zero-event studies. To escape the zero-event problem, Chu et al. [8] excluded the double-zero-event studies from their meta-analyses, which, however, may introduce an estimation bias to the overall effect size [7]. More recently, Xu et al. [13] revisited 442 meta-analyses with or without the double-zero-event studies, and then by a comparative study, they concluded that the double-zero-event studies do contain valuable information and should not be excluded from the meta-analysis.

Inspired by the aforementioned examples, we provide a selective review on the existing methods for meta-analysis that can handle the zero-event studies. For ease of presentation, we will mainly focus on the random-effects model with RR as the effect size, whereas the same comparison also applies to OR and RD. For more details on meta-analysis of OR and RD with the zero-event studies, one may refer to [7] and the references therein, in which the author discussed the methods applicable to all the three effect sizes as well as some methods only applicable to one of them. For a given study, we let *n*_1_ be the number of samples in the treatment group with *X*_1_ being the number of events, and *n*_2_ be the number of samples in the control group with *X*_2_ being the number of events. Let also *X*_1_ follow a binomial distribution with parameters *n*_1_ and *p*_1_>0, and *X*_2_ follow a binomial distribution with parameters *n*_2_ and *p*_2_>0. We further assume that *X*_1_ and *X*_2_ are independent of each other. Then to estimate RR=*p*_1_/*p*_2_, the maximum likelihood estimator is known as

1 $$\begin{array}{@{}rcl@{}} \widehat {\text{RR}} = {X_{1}/n_{1} \over X_{2}/n_{2}} = \frac{X_{1}n_{2}}{X_{2}n_{1}}  \end{array} $$

Note that $\widehat {\text {RR}}$ is often right-skewed. To derive the statistical inference on RR, researchers frequently apply the log scale so that the resulting estimator can be more normally distributed. Specifically by Agresti [14], the approximate variance of $\text {ln}\left (\widehat {\text {RR}}\right)$ is

2 $$\begin{array}{@{}rcl@{}} \text{var}\left[\text{ln}\left(\widehat{\text{RR}}\right)\right]\approx \frac{1}{X_{1}} - \frac{1}{n_{1}} + \frac{1}{X_{2}} - \frac{1}{n_{2}}  \end{array} $$

By (1) and (2), when there are zero events in one or both groups, the classic method for estimating RR suffers from the zero-event problem and will no longer be applicable.

To have a valid estimate of RR, originated from Haldane [15], one often recommends to add 0.5 to the counts of events and non-events if some count is zero [16, 17]. This method is referred to a correction method and has been extensively used in meta-analysis to deal with the zero-event studies. For further developments on the continuity correction, one may refer to Sweeting et al. [18], Carter et al. [19], and the references therein. On the other side, there are also statistical models without the continuity correction to handle meta-analysis with the zero-event studies, such as the generalized linear mixed models [4, 20, 21].

The remainder of this paper is organized as follows. In “Methods with the continuity correction” section, we first review the random-effects model and the existing methods with the continuity correction, and then propose a new method of the continuity correction for estimating RR. In “The generalized linear mixed models” section, we review the generalized linear mixed models for meta-analysis. In “Simulation studies” section, we conduct simulation studies to evaluate the performance of the reviewed methods and our new method. In “Application to COVID-19 data” section, we apply all the well performed methods to a recent meta-analysis on COVID-19 data for further evaluation of their performance. We then conclude the paper in “Discussion” and “Conclusions” sections with some interesting findings, and provide the supplementary materials in the Appendix.

## Methods

### Methods with the continuity correction

Suppose that there are *k* studies in the meta-analysis, and *y*_*i*_ for $i=1, \dots,k$ are the observed effect sizes for each study. By DerSimonian and Laird [22], the random-effects model can be expressed as

3 $$\begin{array}{@{}rcl@{}} y_{i} = \theta +\zeta_{i} + \epsilon_{i}  \end{array} $$

where *θ* is the mean effect size, *ζ*_*i*_ are the deviations of each study from *θ*, and *ε*_*i*_ are the sampling errors. We further assume that *ζ*_*i*_ are independent and identically distributed random variables from *N*(0,*τ*^2^),*ε*_*i*_ are independent random errors from $N(0,\sigma _{i}^{2})$, and that they are independent of each other. In addition, *τ*^2^ is referred to as the between-study variance, and $\sigma _{i}^{2}$ are referred to as the within-study variances.

For the random-effects model in (3), by the inverse-variance method the mean effect size *θ* can be estimated by

4 $$\begin{array}{@{}rcl@{}} \hat{\theta} = \frac{\sum_{i} w^{*}_{i} y_{i}}{\sum_{i} w^{*}_{i}}  \end{array} $$

where $w^{*}_{i} = 1/\left (\sigma _{i}^{2}+ \tau ^{2}\right)$ are the weights assigned to each individual study [23]. In meta-analysis, the within-study variances $\sigma _{i}^{2}$ are routinely estimated by the variances of the observed effect sizes, denoted by var(*y*_*i*_). While for the between-study variance, DerSimonian and Laird [22] proposed the method of moments estimator as

5 $$\begin{array}{@{}rcl@{}} T^{2} = \frac{Q-k+1}{C}  \end{array} $$

where $Q = \sum _{i} w_{i} \left (y_{i} - \sum _{i} w_{i} y_{i} / \sum _{i} w_{i}\right)^{2}$ is known as the *Q* statistic, and $C = \sum _{i} w_{i} - \sum _{i} w_{i}^{2} / \sum _{i} w_{i}$ with $w_{i} = 1/\sigma _{i}^{2}$ for $i=1,\dots,k$.

We note, however, that the random-effects model may suffer from the zero-event problem. Taking RR as an example, if we apply the random-effects model for meta-analysis, then the effect sizes *y*_*i*_ will be the observed ln(RR) values. Now for estimating ln(RR), if we plug in $\widehat {\text {RR}}$ from formula (1) directly, then ln$\left (\widehat {\text {RR}}\right)$ will not be well defined when the studies involve the zero events, and so is for the variance estimate of $\text {ln}\left (\widehat {\text {RR}}\right)$ in formula (2). Consequently, without a valid estimate of the effect size and of its within-study variance, the random-effects model cannot be applied to estimate the mean effect size by the inverse-variance method. This shows that a correction on $\widehat {\text {RR}}$ is often desired in meta-analysis with some studies involving zero events.

#### Existing methods with the continuity correction

Let *c*_1_>0 and *c*_2_>0 be two values for the continuity correction. To overcome the zero-event problem, one common approach is to estimate *p*_1_ by (*X*_1_+*c*_1_)/(*n*_1_+2*c*_1_) and estimate *p*_2_ by (*X*_2_+*c*_2_)/(*n*_2_+2*c*_2_). Plugging them into (1) and (2), we have

6 $$\begin{array}{@{}rcl@{}} \widetilde{\text{RR}}\left(c_{1},c_{2}\right) = {X_{1}+c_{1} \over n_{1}+2c_{1}}\cdot {n_{2}+2c_{2} \over X_{2}+c_{2}}  \end{array} $$

Accordingly, the 95% confidence interval (CI) of RR is

7 $$ \begin{aligned} \text{exp} \left\{{\text{ln}\left(\widetilde{\text{RR}}\left(c_{1},c_{2}\right)\right)} \!\pm\! 1.96\sqrt{ \frac{1}{X_{1}+c_{1}} - \frac{1}{n_{1}+2c_{1}} + \frac{1}{X_{2}+c_{2}} - \frac{1}{n_{2}+2c_{2}}} \right\}  \end{aligned}  $$

For the values of *c*_1_ and *c*_2_ in (6), there are mainly three suggestions in the literature that are widely used for the random-effects meta-analysis.

(i) When *c*_1_=*c*_2_=0.5, it yields the Haldane estimator [15] as
  8 $$ \begin{aligned} \widetilde{\text{RR}}_{\text{Haldane}} = \left\{ \begin{array}{ll} \frac{X_{1}+0.5}{n_{1}+1}\cdot \frac{n_{2}+1}{X_{2}+0.5} & ~~~~~~~~ X_{1}= 0~\text{or}~n_{1}, X_{2}=0~\text{or}~n_{2}, \\ \frac{X_{1}n_{2}}{n_{1}X_{2}} & ~~~~~~~~ \text{otherwise} \end{array} \right. \end{aligned}  $$
(ii) When *c*_1_=*n*_1_/(*n*_1_+*n*_2_) and *c*_2_=*n*_2_/(*n*_1_+*n*_2_), it yields the TACC estimator [18] as
  9 $$ \begin{aligned} \widetilde{\text{RR}}_{\text{TACC}} = \left\{ \begin{array}{ll} \frac{X_{1}+c_{1}}{n_{1}+2c_{1}}\cdot \frac{n_{2}+2c_{2}}{X_{2}+c_{2}} & ~~~~~~~~ X_{1}= 0~\text{or}~n_{1}, X_{2}=0~\text{or}~n_{2}, \\ \frac{X_{1}n_{2}}{X_{2}n_{1}} & ~~~~~~~~ \text{otherwise} \end{array} \right. \end{aligned}  $$
  For the balanced case when *n*_1_=*n*_2_, the TACC estimator is equivalent to the Haldane estimator. Also to implement this estimator, one may apply *metabin* in the R package “meta” with the setting incr=“TACC” [24].
(iii) When *c*_1_=*c*_2_=1, it yields the Carter estimator [19] as
  10 $$\begin{array}{@{}rcl@{}} \widetilde{\text{RR}}_{\text{Carter}} = \frac{X_{1}+1}{n_{1}+2}\cdot \frac{n_{2}+2}{X_{2}+1}  \end{array} $$

Besides the continuity correction methods in family (6), another alternative is to estimate *p*_1_ by (*X*_1_+*c*_1_)/(*n*_1_+*c*_1_) and estimate *p*_2_ by (*X*_2_+*c*_2_)/(*n*_2_+*c*_2_). Then with *c*_1_=*c*_2_=0.5, it yields the Pettigrew estimator [25] as


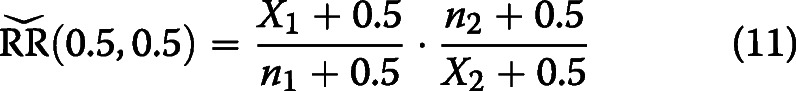
 and the 95% CI of RR as


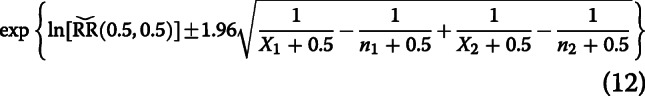


Moreover, to avoid a zero standard error, Hartung and Knapp [26] suggested not to correct *X*_1_ and *X*_2_ when *X*_1_=*n*_1_ and *X*_2_=*n*_2_.

#### A hybrid method with the continuity correction

Note that the existing methods are all constructed to first estimate *p*_1_ and *p*_2_, and then take their ratio as an estimate of RR=*p*_1_/*p*_2_. Nevertheless, noting that *p*_2_ is in the denominator rather than in the numerator, inverting an optimal estimate for *p*_2_ may not necessarily yield an optimal estimate for 1/*p*_2_. In this section, we propose a hybrid method that is to estimate *p*_1_ and 1/*p*_2_ directly, and then take their product to estimate RR.

For the estimation of *p*_1_, we show in Appendix 1 that the mean squared error (MSE) of (*X*_1_+*c*_1_)/(*n*_1_+2*c*_1_) is smaller than the MSE of (*X*_1_+*c*_1_)/(*n*_1_+*c*_1_) in most settings. We thus consider to apply (*X*_1_+*c*_1_)/(*n*_1_+2*c*_1_) to estimate *p*_1_ in RR. While to estimate the reciprocal of *p*_2_, one may consider (*n*_2_+2*c*_2_)/(*X*_2_+*c*_2_) as in (6). Or instead, another option can be to consider (*n*_2_+*c*_2_)/(*X*_2_+*c*_2_) as originated in (??), see also [27] and [28] for more discussion. And if we take the latter one, then a hybrid estimator of RR can be constructed as

11 $$\begin{array}{@{}rcl@{}} \widehat{\text{RR}}\left(c_{1},c_{2}\right) = {X_{1}+c_{1} \over n_{1}+2c_{1}}\cdot {n_{2}+c_{2} \over X_{2}+c_{2}}  \end{array} $$

For the optimal values of *c*_1_ and *c*_2_ in (11), our simulation studies in Appendices 2 and 3 show that *c*_1_=0.5 and *c*_2_=0.5 are among the best options. In view of this, our new hybrid estimator is taken as follows:

12 $$\begin{array}{@{}rcl@{}} \widehat{\text{RR}}(0.5,0.5) = {X_{1}+0.5 \over n_{1}+1}\cdot {n_{2}+0.5 \over X_{2}+0.5}  \end{array} $$

whereas the 95% CI of RR is given as

13 $$ \begin{aligned} \text{exp} \left\{\text{ln}\left(\widehat{\text{RR}}(0.5,0.5)\right) \!\pm\! 1.96\sqrt{ \frac{1}{X_{1}+0.5} - \frac{1}{n_{1}+1} + \frac{1}{X_{2}+0.5} - \frac{1}{n_{2}+0.5}} \right\}  \end{aligned}  $$

#### Comparison of the continuity correction methods

In this section, we conduct a numerical study to compare the finite sample performance of the existing and new methods. For ease of presentation, we refer to the confidence intervals associated with (8), (9), (10), (??) and (12) as the Haldane interval, the TACC interval, the Carter interval, the Pettigrew interval, and the hybrid interval, respectively.

To generate the data, we let *p*_2_=0.05, 0.15, 0.85 or 0.95, and *p*_1_=*p*_2_×RR with RR ranging from 0.2 to min{5,1/*p*_2_}. We also consider different combinations of the sample sizes. For the sake of brevity, only the results for balanced samples with *n*_1_=*n*_2_=10 or 50 are presented, whereas the results for the unbalanced samples are postponed to Appendix 4. Recall that the Haldane and TACC intervals are the same when *n*_1_=*n*_2_, and we thus present the results for the Haldane interval only. With *N*=100,000 repetitions for each setting, we generate random numbers from the binomial distributions with parameters (*p*_1_,*n*_1_) and (*p*_2_,*n*_2_) to yield the estimates of RR and their CIs. We then compute the frequencies of the true RR falling in the CIs as the coverage probability estimates. Moreover, the expected lengths of the CIs on the log scale are computed by $N^{-1}\sum _{s=1}^{N}\left (\text {ln(UL}_{\text {s}}) - \text {ln(LL}_{\text {s}})\right)$, where UL_*s*_ and LL_*s*_ are the upper and lower limits of the *s*th CI.

**Fig. 1:**
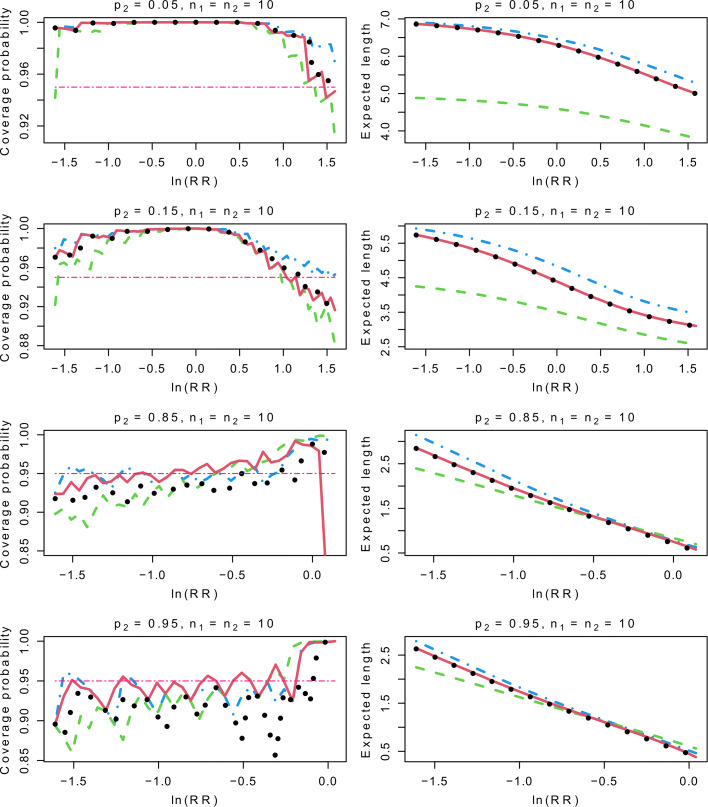
Comparison of the four CIs of RR with *p*_2_=0.05, 0.15, 0.85 or 0.95, and *n*_1_=*n*_2_=10. The dot-dashed lines represent the simulation results of the Haldane interval, the dashed lines represent the simulation results of the Carter interval, the dotted lines represent the simulation results of the Pettigrew interval, and the solid lines represent the simulation results of the hybrid interval. CI: Confidence interval, RR: Relative risk

**Fig. 2:**
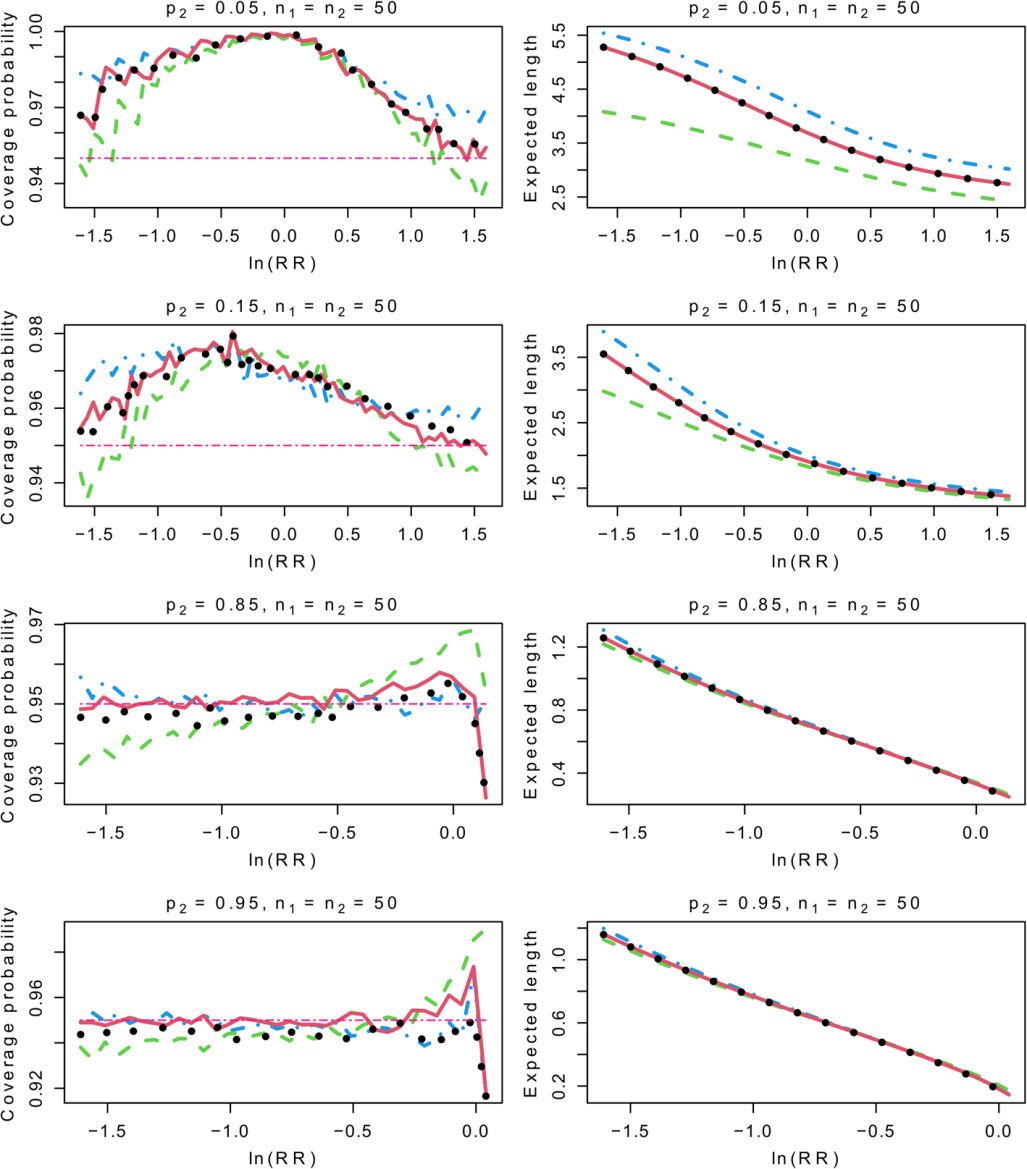
Comparison of the four CIs of RR with *p*_2_=0.05, 0.15, 0.85 or 0.95, and *n*_1_=*n*_2_=50. The dot-dashed lines represent the simulation results of the Haldane interval, the dashed lines represent the simulation results of the Carter interval, the dotted lines represent the simulation results of the Pettigrew interval, and the solid lines represent the simulation results of the hybrid interval. CI: Confidence interval, RR: Relative risk

For *p*_2_=0.05 or 0.15, the top four panels of Figs. 1 and 2 show that the Haldane interval is the most conservative interval in most settings, and it provides the longest expected lengths compared to the other three intervals. The Carter interval may have downward spikes in the left or right tail, although it leads to the shortest expected lengths. We also note that the simulation results of the Pettgrew interval and the hybrid interval are nearly the same. Their coverage probabilities and expected lengths are intermediate between those of the other two intervals in most settings.

From the bottom four panels of Figs. 1 and 2 with *p*_2_=0.85 or 0.95, it is evident that the Haldane interval has a satisfactory performance in most settings with the coverage probabilities around the nominal level. In contrast, the Carter interval fails to provide enough large coverage probabilities in most settings, so does the Pettgrew interval when *n*_1_ and *n*_2_ are small. Note also that the coverage probabilities of the hybrid interval are comparable to the Haldane interval as long as *p*_2_ is not extremely large. Moreover, the hybrid interval yields shorter expected lengths than the Haldane interval.

To sum up, when *p*_2_ is small, the Pettgrew interval and the hybrid interval are less conservative than the Haldane interval in most settings. While for large *p*_2_, the Haldane interval and the hybrid interval perform better than the Pettgrew interval in terms of coverage probability. In addition, the expected lengths of the hybrid interval are always shorter than the Haldane interval. This shows that the hybrid interval can serve as a good alternative for the interval estimation of RR.

### The generalized linear mixed models

The generalized linear mixed models (GLMMs) are extensions of the generalized linear model, which include both the fixed and random effects as linear predictors [14]. Different types of the GLMMs have been proposed in the literature including a few reviews and comparison studies [4, 29]. Among the existing models, the bivariate GLMM has been well recognized and being recommended for estimating RR in meta-analysis [20].

Let *p*_*i*1_ and *p*_*i*2_ be the event probabilities in the treatment and control groups of the *i*th study, respectively. The bivariate GLMM is represented as

14 $$\begin{array}{@{}rcl@{}} &&g(p_{i1}) = \Omega_{1} + \zeta_{i1} \\ &&g(p_{i2}) =\Omega_{2} + \zeta_{i2} \end{array} $$

where *g*(·) is the link function, *Ω*_1_ and *Ω*_2_ are the fixed effects, and the random effects are given by

$$\begin{array}{@{}rcl@{}} { \left(\begin{array}{c} \zeta_{i1} \\ \zeta_{i2} \end{array} \right)} \overset{\text{ind}}{\sim} { N\left[ \left(\begin{array}{c} 0 \\ 0 \end{array} \right), \left(\begin{array}{cc} \tau_{1}^{2} & \rho \tau_{1} \tau_{2} \\ \rho \tau_{1} \tau_{2} & \tau_{2}^{2} \end{array} \right) \right ]} \end{array} $$

The mean effect size based on model (14) was defined as

15 $$\begin{array}{@{}rcl@{}} {}{\text{RR}}_{\text{GLMM}} =\frac{E\left(p_{1}\right)}{E\left(p_{2}\right)} = \frac{\int_{-\infty}^{\infty}g^{-1}\left(\Omega_{1}+t\right)\tau_{1}^{-1} \phi\left(t/\tau_{1}\right) \mathrm{d}t}{\int_{-\infty}^{\infty}g^{-1}\left(\Omega_{2}+t\right)\tau_{2}^{-1} \phi\left(t/\tau_{2}\right) \mathrm{d}t}  \end{array} $$

where *E*(*p*_1_) and *E*(*p*_2_) are the mean event probabilities in the control and treatment groups, *g*^−1^(·) is the inverse function of the link, and *ϕ*(·) is the probability density function of the standard normal distribution [30]. For the logit link, Zeger et al. [31] proposed an approximate formula $E\left (p_{j}\right)\approx \text {expit}\left (\Omega _{j} /\sqrt {1+C^{2}\tau _{j}^{2}}\right)$ with $C = 16\sqrt {3}/(15\pi)$. For the probit link, $E\left (p_{j}\right)=\Phi \left (\Omega _{j} /\sqrt {1+\tau _{j}^{2}}\right)$, where *j*=1 or 2, and *Φ*(·) is the cumulative distribution function of the standard normal distribution. While for the other links, there does not exist a closed form of formula (15) and so a numerical approximation is often needed [32].

For the parameter estimation in model (14), Jackson et al. [4] provided a detailed introduction for the implementation based on the R package “lme4” in their model 6. Alternatively, one may also apply the function *meta.biv* in the R package “altmeta” maintained by Lin and Chu [33], in which the 95% CI of RR can be derived by the bootstrap resampling method.

## Results

### Simulation studies

In this section, we compare the performance of the reviewed methods on handling meta-analysis with the zero-event studies, including the continuity correction methods and the generalized linear mixed models. Among the existing continuity correction methods, we note that the Haldane and TACC estimators are comparable and among the best when estimating the mean effect size, in contrast to the other two methods including the Carter and Pettigrew estimators. Hence, for the sake of brevity, we only present the results of the Haldane and TACC estimators in the main text but provide the simulation results for all four methods in Appendix 5. Besides the Haldane and TACC estimators, we also consider the newly introduced hybrid estimator and the GLMM with the logit link for further comparison.

To conduct the meta-analysis, we consider *k*=3, 6 and 12 as three different numbers of studies. Also by (3), we let *θ*=ln(RR) be the mean effect size that ranges from ln(0.2) to ln(5), and then generate the random effects *ζ*_*i*_ from *N*(0,*τ*^2^) with *τ*^2^= 0.25 or 1. Next, we randomly generate *n*_*i*2_ from the log-normal distribution based on the assumption that ${\ln }(n_{i2}) \overset {\text {ind}}{\sim } N(3.35, 1.00)$ [34]. It is also assumed by [34] that the ratios between *n*_*i*1_ and *n*_*i*2_ follow the uniform distribution with values from 0.84 to 2.04. In addition, we generate the event probabilities of the control group *p*_*i*2_ from the uniform distribution with values from 0.01 to min{0.99,1/exp(*θ*)}. Then accordingly, the event probabilities of the treatment group are given by *p*_*i*1_=exp(*θ*+*ζ*_*i*_)*p*_*i*2_, where exp(*θ*+*ζ*_*i*_)*p*_*i*2_≥1 will be discarded. Finally, we generate *X*_*i*1_ and *X*_*i*2_ from the binomial distributions with parameters (*n*_*i*1_,*p*_*i*1_) and (*n*_*i*2_,*p*_*i*2_), respectively. Note that the data will be re-generated if the number of events or non-events in one group are both zero. Finally, with *N*=10,000 repetitions for each setting, we compute the mean squared errors (MSEs) between the estimated RR and the true RR to evaluate the accuracy of the methods.

**Fig. 3:**
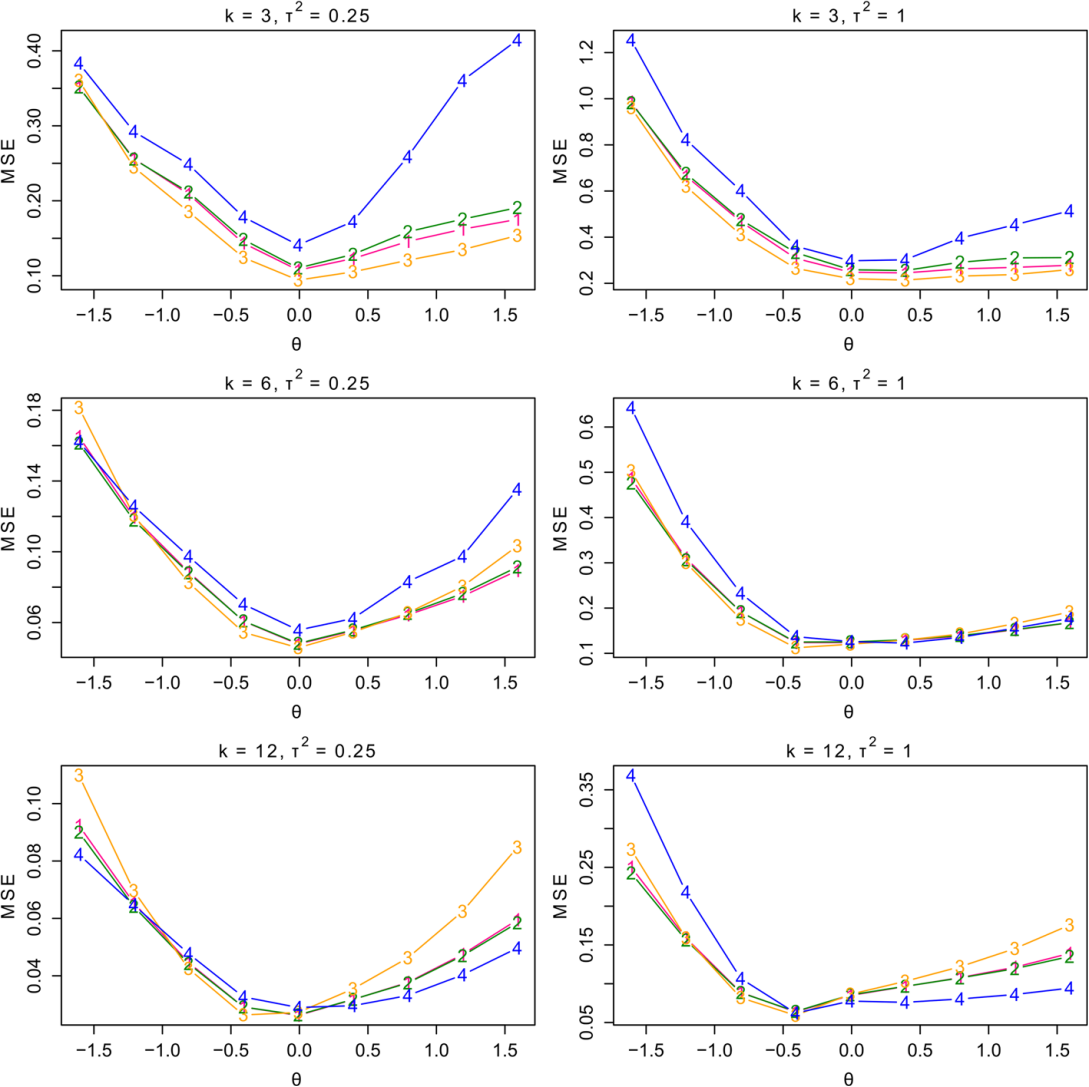
Comparison of the four methods with *k*=3, 6 or 12, *τ*^2^=0.25 or 1. “1” represents the results of the random-effects model with the Haldane estimator, “2” represents the results of the random-effects model with the TACC estimator, “3” represents the results of the random-effects model with the hybrid estimator, and “4” represents the results of the GLMM. TACC: Treatment arm continuity correction, GLMM: Generalized linear mixed model, MSE: Mean squared error

From the top two panels of Fig. 3, it is evident that the three continuity correction methods perform much better than the GLMM in nearly all settings when *k* is small. Moreover, the hybrid estimator is consistently better than the Haldane and TACC estimators. The middle two panels show that, when *k* is moderate, the three continuity correction methods still perform better than the GLMM in most settings. Finally, the bottom two panels indicate that the GLMM performs the best in most settings when *k* is large. To conclude, the accuracy of the different methods depends on the number of studies. In particular, for meta-analysis with few studies, the random-effects model with the hybrid estimator is more reliable for handling the zero-event studies than the other methods; and for meta-analysis with large studies, we recommend the GLMM to handle the random-effects meta-analysis.

### Application to COVID-19 data

As mentioned earlier, Chu et al. [8] conducted a systematic review that revealed the connections of physical distancing, face masks, and eye protection with the transmission of SARS, MERS, and COVID-19. It is noteworthy that their analytical results have attracted more and more attention. As an evidence, their paper has received a total of 1236 citations in Google Scholar as of 16 March 2021. In this section, we propose to reanalyze COVID-19 data and compare the performance of the different methods with or without the double-zero-event studies, including the Haldane estimator, the TACC estimator, the hybrid estimator, and the GLMMs.

**Fig. 4:**
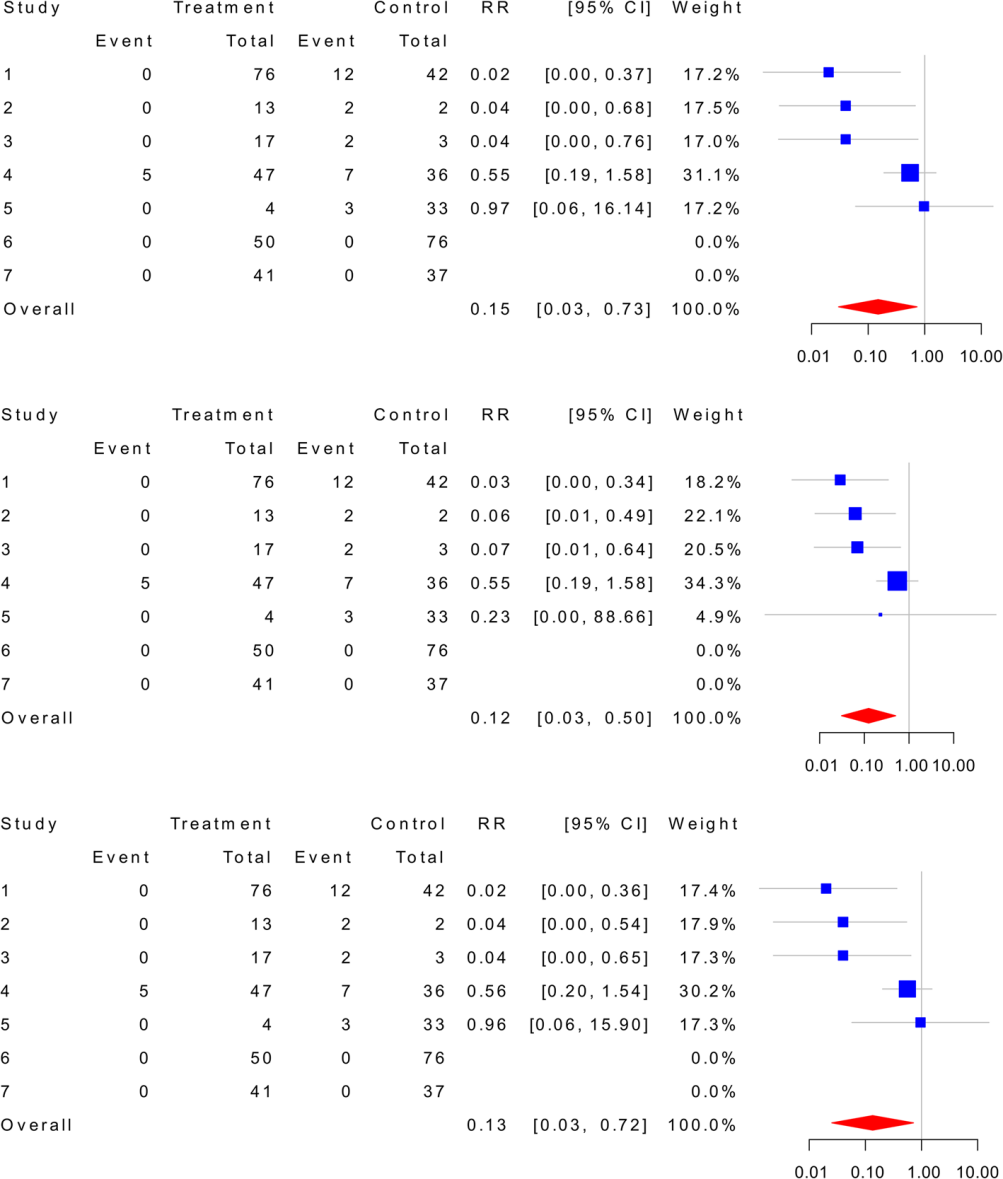
Meta-analyses of COVID-19 data without the double-zero-event studies by applying the Haldane estimator (top), the TACC estimator (middle), and the hybrid estimator (bottom). COVID-19: Coronavirus disease 2019, TACC: Treatment arm continuity correction, RR: Relative risk, CI: Confidence interval

Note that the treatment group represents a further physical distance and the control group represents a shorter physical distance. As shown in the top panel of Fig. 4, [8] applied the random-effects model with the Haldane estimator and removed the double-zero-event studies from their meta-analysis. The overall effect size of 0.15 with the 95% CI being [0.03,0.73] indicates that the infection risk will be significantly reduced with a further physical distance. The middle panel of Fig. 4 reports that the random-effects model with the TACC estimator yields the overall effect size of 0.12 with the 95% CI being [0.03,0.50]. Moreover, the bottom panel of Fig. 4 shows that the random-effects model with the hybrid estimator yields the overall effect size of 0.13 with the 95% CI being [0.03,0.72]. Note also that the study-specific CIs here are always narrower than the CIs in the top panel, which coincides with the simulation results that the expected lengths of the CI associated with the hybrid estimator are shorter than the Haldane estimator. In addition, the GLMM in (14) does not provide the estimates of the study-specific effect sizes, so the results are listed as follows. By the bootstrap resampling with 1000 replicates, the GLMM with the logit link yields the overall effect size of 0.20 with the 95% bootstrap CI being [0.05,0.55]. Also, the GLMM with the probit link yields the overall effect size of 0.18 with the 95% CI being [0.04,0.55].

**Fig. 5:**
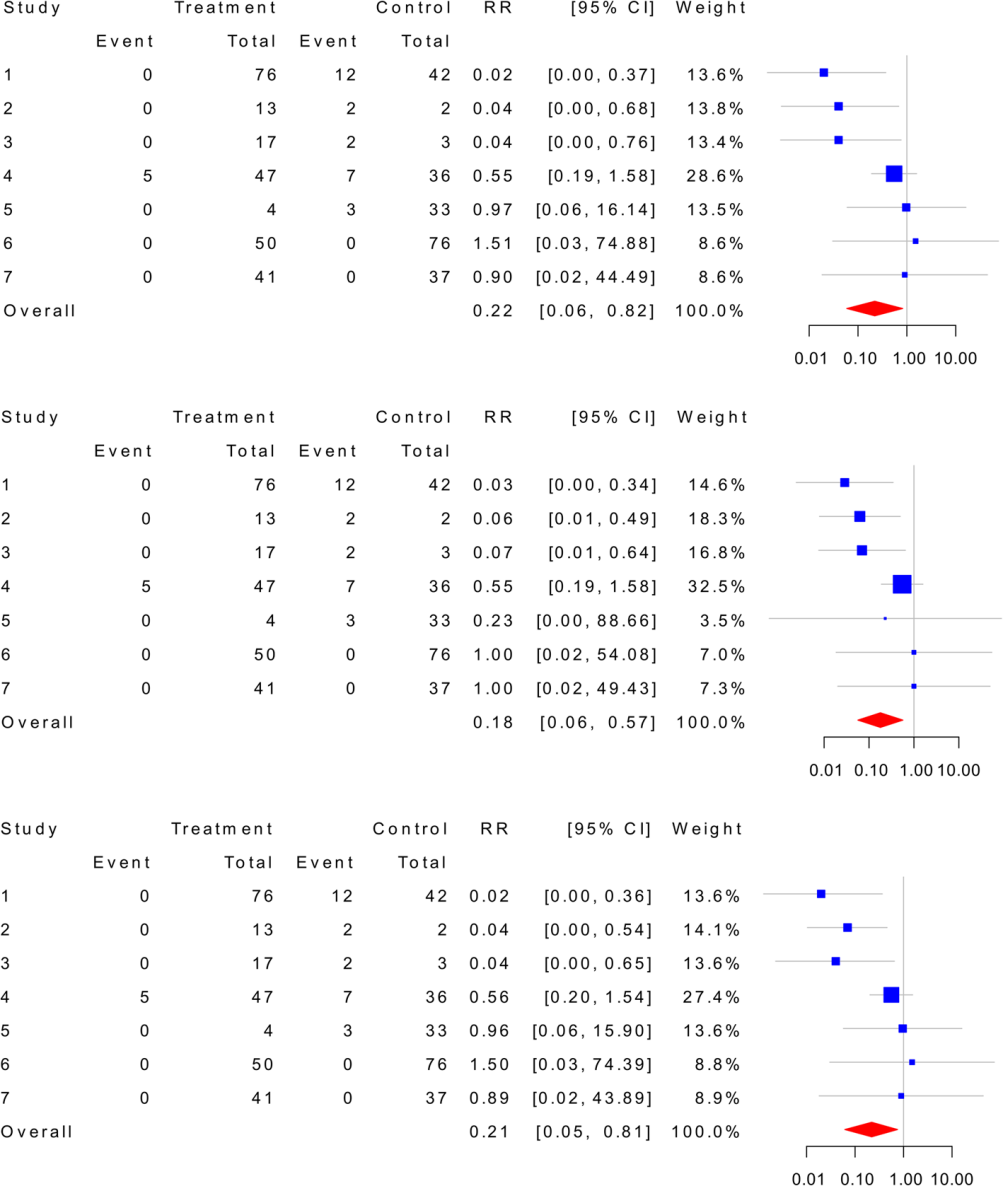
Meta-analyses of COVID-19 data with the double-zero-event studies by applying the Haldane estimator (top), the TACC estimator (middle), and the hybrid estimator (bottom). COVID-19: Coronavirus disease 2019, TACC: Treatment arm continuity correction, RR: Relative risk, CI: Confidence interval

To reanalyze COVID-19 data, we now include the double-zero-event studies. The top panel of Fig. 5 shows that the random-effects model with the Haldane estimator yields the overall effect size of 0.22 with 95% CI being [0.06,0.82]. The middle panel of Fig. 5 presents that the random-effects model with the TACC estimator provides the overall effect size of 0.18 with the 95% CI being [0.06,0.57]. While for the hybrid estimator, it is shown by the bottom panel that the overall effect size is 0.21 with 95% CI being [0.05, 0.81]. At last, the GLMM with the logit link provides the overall effect size of 0.29 with the 95% CI being [0.10,0.64], and the GLMM with the probit link provides the overall effect size of 0.28 with the 95% CI being [0.10,0.56].

## Discussion

To handle the zero-event studies in meta-analysis of binary data, researchers often apply the random-effects model with the continuity correction, or instead, the GLMMs. From the simulation results, we note that the performance of the different methods depends on the number of studies. For meta-analysis with few studies, the random-effects model with the continuity correction is able to perform better than the GLMM, especially the hybrid continuity correction. We also note that the hybrid continuity correction can yield a reliable confidence interval for a single RR. Although the continuity correction does show some advantages, it should be used with caution since an arbitrary correction may lead to a bias or even reverse the result of a meta-analysis, especially when the numbers of samples in the two groups are fairly unbalanced [7, 13]. When the number of studies is large, the GLMM is preferable to the random-effects model with the continuity correction. In other words, the performance of the GLMM relies on a sufficient number of studies [35]. Also as shown in Ju et al. [34], the GLMM also requires enough total events in the two groups, e.g., larger than 10.

Besides the random-effects model we have compared, it is noteworthy that there are also other models for meta-analysis that can handle the zero-event studies including, for example, the beta-binomial model [36–38]. Most meta-analyses with rare events have a small degree of heterogeneity, and so the common-effect model may be more suitable than the random-effects model [39]. In addition, Li and Rice [40] showed that the fixed-effects model can also provide an accurate CI for meta-analysis of OR with the zero-event studies. Apart from that, it is also noteworthy that the fixed-effects model can serve as a convincing model for meta-analysis with few studies [12, 41–43]. As a future work, it can be interesting to investigate the best model for meta-analysis with few studies which include the zero-event studies as well.

For the double-zero-event studies in meta-analysis, we have shown by reanalyzing COVID-19 data that they do impact the estimate of the mean effect size, and so they may not be uninformative. As noted by Friedrich et al. [44], including the double-zero-event studies moves the mean effect size estimate toward the direction of the null hypothesis. If one arbitrarily excluded the informative double-zero-event studies, there would be a risk of overstating the treatment effect such that the conclusion would be less reliable. As recommended by the literature [7, 13] and the references therein, we suggest including the double-zero-event studies in meta-analysis.

Apart from model comparison, the selection of effect sizes has attracted more and more attention in the literature. In particular, there is a recent debate on the choice of RR or OR in clinical epidemiology, in which a number of important properties of RR or OR together with their pros and cons were discussed including, for example, portability and collapsibility [45–47]. In view of this, we have also analyzed COVID-19 data with OR being the effect size and present the results in Appendix 6 with R code in Appendix 7. To handle the zero-event studies, we apply four methods that have been reviewed in this paper, including Haldane’s continuity correction, TACC, the GLMM, and the empirical continuity correction proposed by Sweeting et al. [18]. For more techniques on meta-analysis of OR with the zero-event studies, one may refer to [4, 7, 18, 29, 34] and the references therein.

## Conclusions

In this paper, we revisited the existing methods that are widely used to handle the zero-event problem in meta-analysis of binary data, in particular with RR as the effect size which is also known as the risk ratio. For the methods with the continuity correction, we reviewed four existing estimators of RR and also introduced a new hybrid estimator with their applications to the random-effects model. Apart from those, the GLMM was also included which is a state-of-the-art method without the continuity correction. By a comparative study and also a real data analysis on COVID-19 data, we found that the random-effects model with the hybrid estimator can serve as a more reliable method for handling the zero-event studies when there are few studies in a meta-analysis, and recommend using the GLMM when the number of studies is large. This paper also provides a useful addition to Chu et al. [8], and meanwhile, it also calls for further observational studies in this field.

## Supplementary Information

**Additional file 1** Supplementary information: Appendix

## Data Availability

Not applicable.

## Abbreviations

OR: Odds ratio
RR: Relative risk
RD: Risk difference
SARS: Severe acute respiratory syndrome
MERS: Middle East respiratory syndrome
COVID-19: Coronavirus disease 2019
CI: Confidence interval
MSE: Mean squared error
GLMMs: Generalized linear mixed models
TACC: Treatment arm continuity correction
