# Supplementary material for: Meta-analysis with zero-event studies: a comparative study with application to COVID-19 data
Source: Mil Med Res. 2021 Jul 3;8:41. doi: 10.1186/s40779-021-00331-6 (PMC8254431; doi:10.1186/s40779-021-00331-6)
Supplement: Supplementary file 1 — Additional file 1 Supplementary information: Appendix [file 40779_2021_331_MOESM1_ESM.pdf]

Appendix

Appendix 1

Note that X_1 is a random variable from the binomial distribution with parameters n_1 and p_1, and so we have

$$\begin{aligned}
\text{Bias}\left(\frac{X_1+c_1}{n_1+2c_1}\right) &= \text{E}\left(\frac{X_1+c_1}{n_1+2c_1}\right) - p_1 \\
&= \frac{n_1p_1+c_1}{n_1+2c_1} - \frac{n_1p_1+2c_1p_1}{n_1+2c_1}, \\
&= \frac{c_1-2c_1p_1}{n_1+2c_1}; \\
\text{Bias}\left(\frac{X_1+c_1}{n_1+c_1}\right) &= \text{E}\left(\frac{X_1+c_1}{n_1+c_1}\right) - p_1 \\
&= \frac{n_1p_1+c_1}{n_1+c_1} - \frac{n_1p_1+c_1p_1}{n_1+c_1}, \\
&= \frac{c_1-c_1p_1}{n_1+c_1}.
\end{aligned}$$

Then we propose to solve

$$\left(\frac{c_1-2c_1p_1}{n_1+2c_1}\right)^2 < \left(\frac{c_1-c_1p_1}{n_1+c_1}\right)^2.$$

For $p_1 \leqslant 0.5$, it is evident that this inequality always holds. For $p_1 > 0.5$, we have

$$\begin{aligned}
\frac{2c_1p_1-c_1}{n_1+2c_1} &< \frac{c_1-c_1p_1}{n_1+c_1} \\
2c_1p_1n_1+2c_1^2p_1-c_1n_1-c_1^2 &< c_1n_1-c_1n_1p_1+2c_1^2-2c_1^2p_1 \\
3c_1p_1n_1+4c_1^2p_1 &< 2c_1n_1+3c_1^2 \\
p_1 &< \frac{2n_1+3c_1}{3n_1+4c_1}.
\end{aligned}$$

Since $(2n_1+3c_1)/(3n_1+4c_1) > 0.5$, the squared bias of $(X_1+c_1)/(n_1+2c_1)$ is lower than that of $(X_1+c_1)/(n_1+c_1)$ over a half of settings. We also note that the variance of $(X_1+c_1)/(n_1+2c_1)$ is always smaller than the variance of $(X_1+c_1)/(n_1+c_1)$. Hence,

the MSE of $(X_1 + c_1)/(n_1 + 2c_1)$ is smaller than the MSE of $(X_1 + c_1)/(n_1 + c_1)$ in most settings.

Appendix 2: Comparison of the p_1 estimates

In this simulation study, we propose to explore the effect of c_1 on the family of estimators $\tilde{p}_1(c_1) = (X_1 + c_1)/(n_1 + 2c_1)$ in terms of coverage probability and expected length. To generate the simulation data, we let p_1 range from 0.01 to 0.99 and $c_1 = 0.25$, 0.5, 0.75 or 1. We also consider $n_1 = 10$ and 50 as the different numbers of samples. With $N = 100,000$ repetitions for each setting, we generate random numbers from the binomial distribution with parameters p_1 and n_1 to yield the estimates of p_1 and the CIs. By the delta method, the variance of $\ln[\tilde{p}_1(c_1)]$ can be approximately given as $\text{var}[\ln(\tilde{p}_1(c_1))] \approx 1/(X_1 + c_1) - 1/(n_1 + 2c_1)$. Hence, the 95% confidence interval of p_1 is given by

$$\exp\left\{\ln\left(\frac{X_1 + c_1}{n_1 + 2c_1}\right) \pm 1.96\sqrt{\frac{1}{X_1 + c_1} - \frac{1}{n_1 + 2c_1}}\right\}. \tag{1}$$

Then we compute the frequencies of the true RR falling in the CIs as the coverage probability estimates. The expected lengths of the CIs on the log scale are computed by $N^{-1}\sum_{s=1}^{N}[\ln(\text{UL}_\text{s}) - \ln(\text{LL}_\text{s})]$, where UL_s and LL_s are the upper and lower limits for the sth CI.

From Additional Fig. 1, it is evident that the CIs with small c_1, e.g. $c_1 = 0.25$, yield low coverage probabilities when p_1 is close to 1. On the other side, the CIs with large c_1, e.g. $c_1 = 1$, have low coverage probabilities when p_1 is close to 0. In addition, the CIs with larger c_1 will yield shorter expected lengths, but large c_1 may harm the coverage probabilities for small p_1. As a compromise, we recommend the intermediate value $c_1 = 0.5$, and our subsequent results show that $c_1 = 0.5$ is indeed a reliable option for estimating RR.

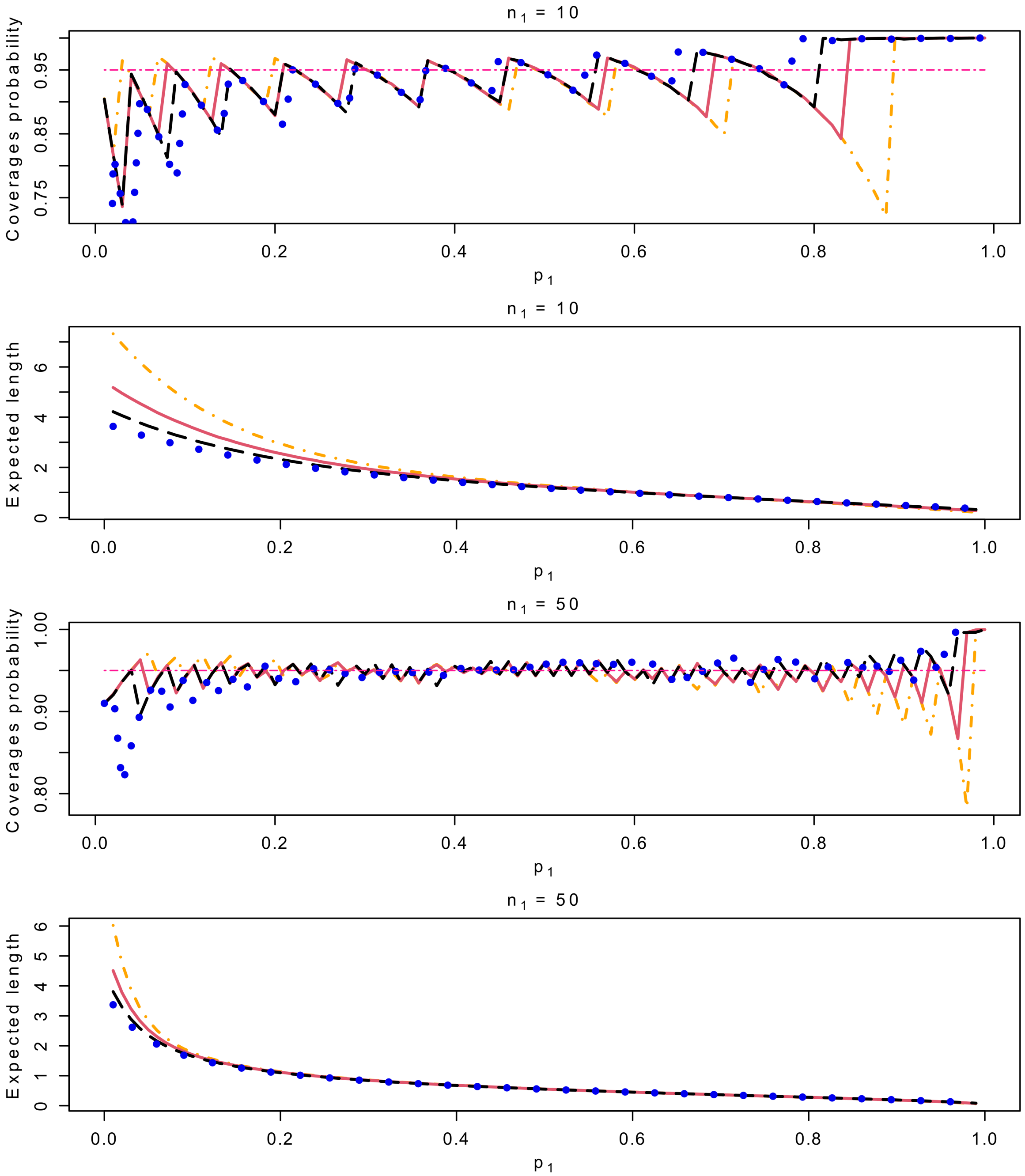

Additional Fig. 1: Comparison of the CIs of p_1 with $c_1 = 0.25$, 0.5, 0.75 or 1, and $n_1 = 10$ or 50. The dot-dashed lines represent the simulation results for $c_1 = 0.25$, the solid lines represent the simulation results for $c_1 = 0.5$, the dashed lines represent the simulation results for $c_1 = 0.75$, and the dotted lines represent the simulation results for $c_1 = 1$. CI: Confidence interval

Appendix 3: Simulation study for the estimators within family (13)

By the results from Appendix 2, we let $c_1 = 0.5$ and consider $c_2 = 0.5$ or 1 for estimator (6) and (13). To generate the simulation data, we let $p_2 = 0.05$, 0.15, 0.85 or 0.95, and $p_1 = p_2 \times \text{RR}$ with RR ranging from 0.2 to $\min\{5, 1/p_2\}$. We also consider the numbers of samples as $n_1 = n_2 = 10$ or 50. With $N = 100,000$ repetitions for each setting, we generate random numbers from the binomial distributions with parameters (p_1, n_1) and (p_2, n_2). We then compute the frequencies of the true RR falling in the CIs as the coverage probability estimates. The expected lengths of the CIs on the log scale are computed by $N^{-1}\sum_{s=1}^{N}[\ln(\text{UL}_\text{s}) - \ln(\text{LL}_\text{s})]$, where UL_s and LL_s are the upper and lower limits of the sth CI.

From the top four panels of Additional Fig. 2 and 3 with small p_2, the CIs associated with $\widetilde{\text{RR}}(0.5, 1)$ and $\widehat{\text{RR}}(0.5, 1)$ yield shorter expected lengths than the other two CIs, but they have low coverage probabilities when ln(RR) is large. By contrast, the CIs associated with $\widetilde{\text{RR}}(0.5, 0.5)$ and $\widehat{\text{RR}}(0.5, 0.5)$ are able to provide a better performance for large ln(RR). From the bottom four panels of Additional Fig. 2 and 3 with large p_2, we note that the CIs associated with $\widehat{\text{RR}}(0.5, 0.5)$ and $\widehat{\text{RR}}(0.5, 1)$ perform better in terms of coverage probability in most settings. Noting also that the expected lengths of the four CIs are almost the same, we thus conclude that the CI associated with $\widehat{\text{RR}}(0.5, 0.5)$ is the best among the four CIs.

Appendix 4: Simulation study for unbalanced n_1 and n_2

In this simulation study, we compare the performance of the four existing intervals and the hybrid interval for unbalanced n_1 and n_2. Specifically, we consider $n_1 = 20$ with $n_2 = 40$, 80 or 160, and consider $n_2 = 20$ with $n_1 = 40$, 80 or 160. The other settings are kept the same as those in the main text.

From the top four panels of Additional Fig. 4 to 9 with small p_2, the Haldane and TACC intervals are more stable than the other CIs in terms of coverage probability. We

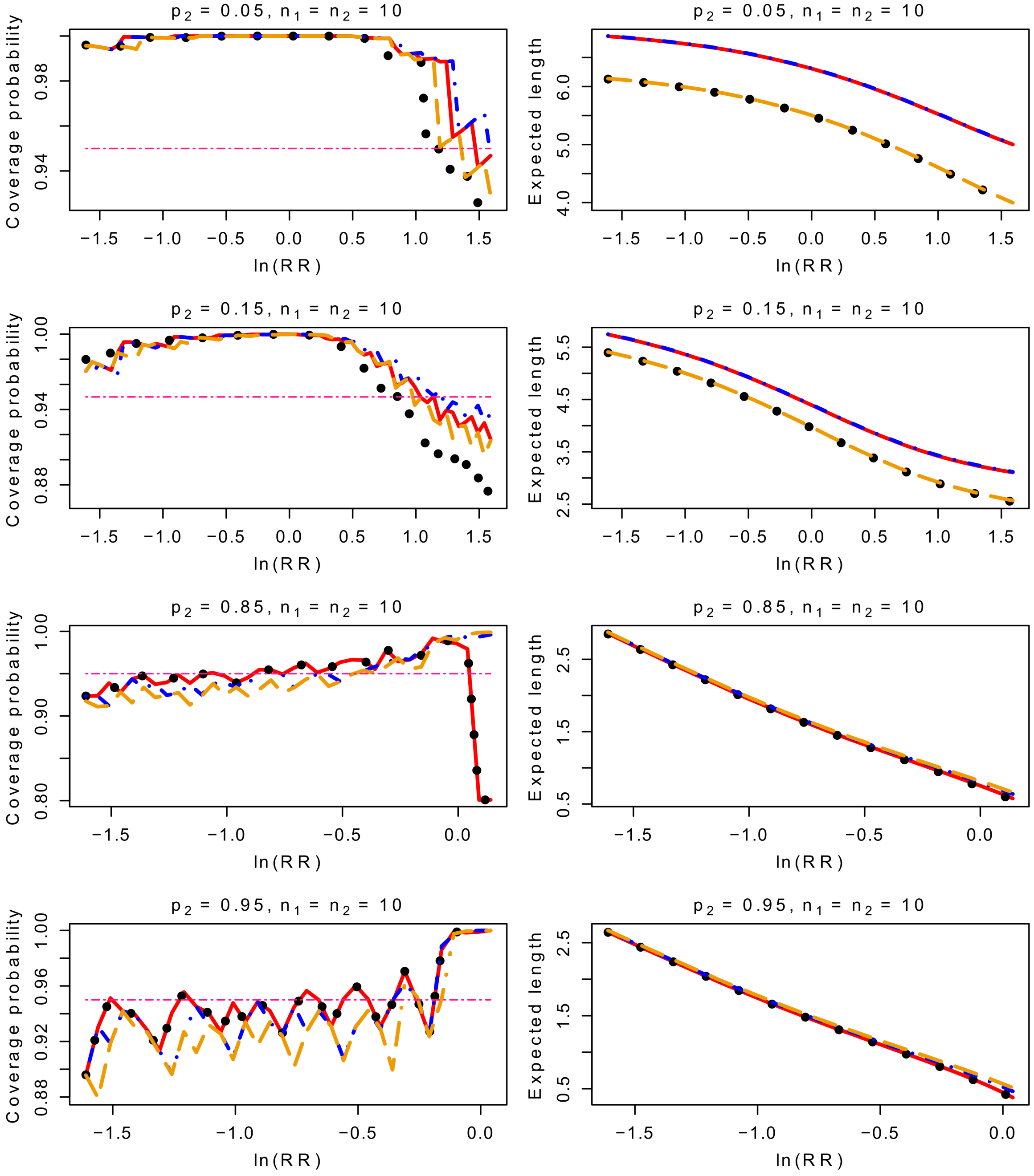

Additional Fig. 2: Comparison of the four CIs of RR with $p_2 = 0.05$, 0.15, 0.85 or 0.95, and $n_1 = n_2 = 10$. The dot-dashed lines represent the simulation results of the CI associated with $\widetilde{\mathrm{RR}}(0.5, 0.5)$, the dashed lines represent the simulation results of the CI associated with $\widetilde{\mathrm{RR}}(0.5, 1)$, the solid lines represent the simulation results of the CI associated with $\widehat{\mathrm{RR}}(0.5, 0.5)$, and the dotted lines represent the simulation results of the CI associated with $\widehat{\mathrm{RR}}(0.5, 1)$. CI: Confidence interval, RR: Relative risk

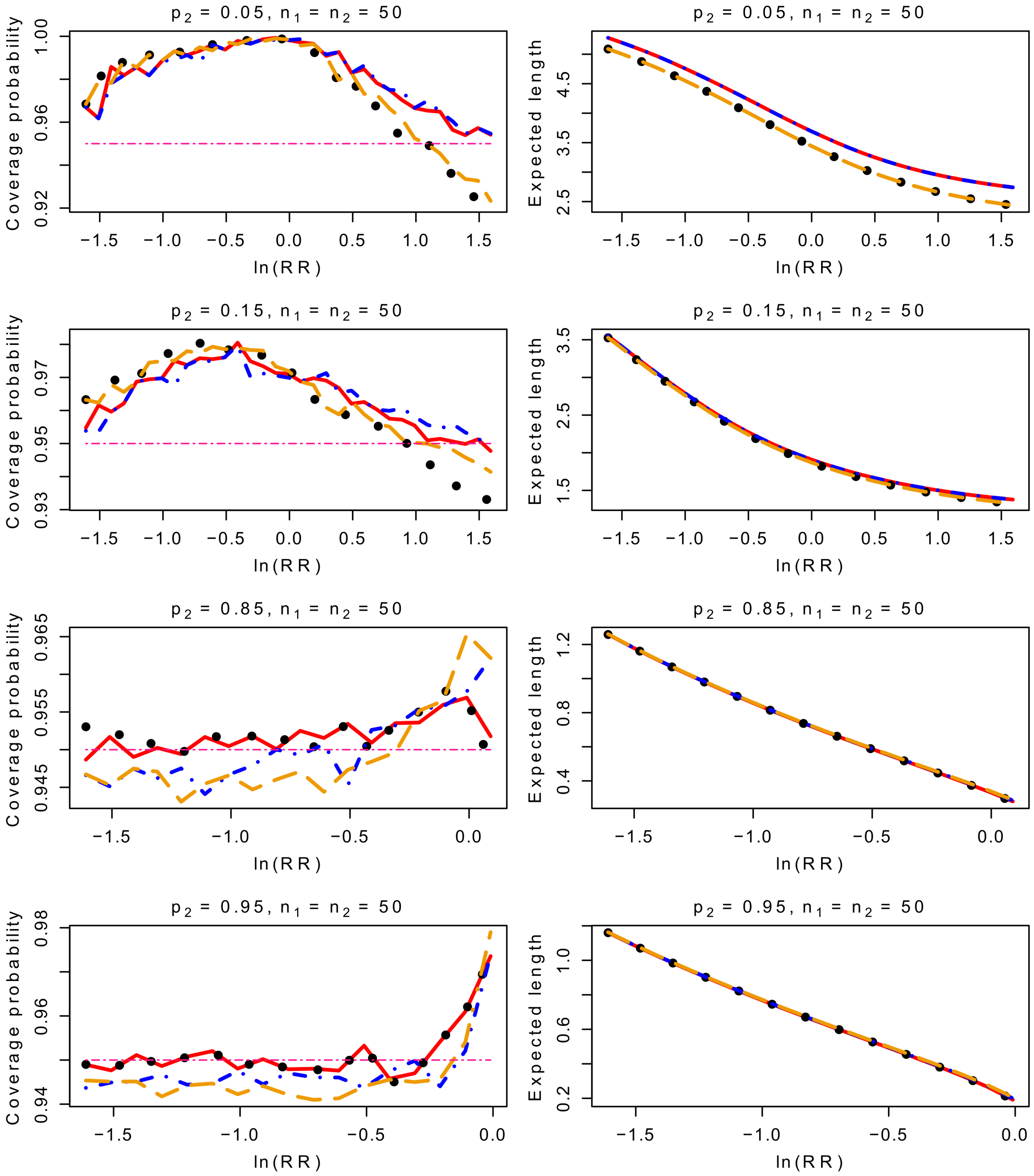

Additional Fig. 3: Comparison of the four CIs of RR with $p_2 = 0.05$, 0.15, 0.85 or 0.95, and $n_1 = n_2 = 50$. The dot-dashed lines represent the simulation results of the CI associated with $\widetilde{\mathrm{RR}}(0.5, 0.5)$, the dashed lines represent the simulation results of the CI associated with $\widetilde{\mathrm{RR}}(0.5, 1)$, the solid lines represent the simulation results of the CI associated with $\widehat{\mathrm{RR}}(0.5, 0.5)$, and the dotted lines represent the simulation results of the CI associated with $\widehat{\mathrm{RR}}(0.5, 1)$. CI: Confidence interval, RR: Relative risk

also note that the expected lengths of the Haldane interval are much shorter than the TACC interval. From the bottom four panels of Additional Fig. 4 to 9 with large p_2, the hybrid interval provides the best performance with the coverage probabilities close to the nominal level when $n_1 < n_2$. When $n_1 > n_2$, the CIs except the Carter interval are almost identical in most settings, as long as ln(RR) is not very large.

Appendix 5: Comparison of the random-effects models with the existing correction methods

Appendix 6: Meta-analyses of COVID-19 data with OR being the effect size

By applying Haldane's continuity correction, we note from the top panel of Additional Fig. 11 that the overall effect size of 0.09 with the 95% CI being $[0.01, 0.67]$ indicates a significant effect of a further physical distance. The middle panel of Additional Fig. 11 shows that the random-effects model with the treatment arm continuity correction yields the overall effect size of 0.07 with the 95% CI being $[0.01, 0.54]$. Moreover, the bottom panel of Additional Fig. 11 shows that the random-effects model with the empirical continuity correction yields the overall effect size of 0.06 with the 95% CI being $[0.01, 0.51]$. In addition, for the results not presented in Additional Fig. 11, the GLMM with the logit link yields the overall effect size of 0.15 with the 95% bootstrap CI being $[0.02, 0.51]$. Meanwhile, the GLMM with the probit link yields the overall effect size of 0.14 with the 95% CI being $[0.01, 0.51]$.

After including the double-zero-event studies, the top panel of Additional Fig. 12 shows that the random-effects model with Haldane's continuity correction yields the overall effect size of 0.16 with 95% CI being $[0.03, 0.82]$. The middle panel of Additional Fig. 12 presents that the random-effects model with the treatment arm continuity correction yields the overall effect size of 0.14 with the 95% CI being $[0.03, 0.68]$. Then for the empirical continuity correction, we note from the bottom panel of Additional Fig. 12

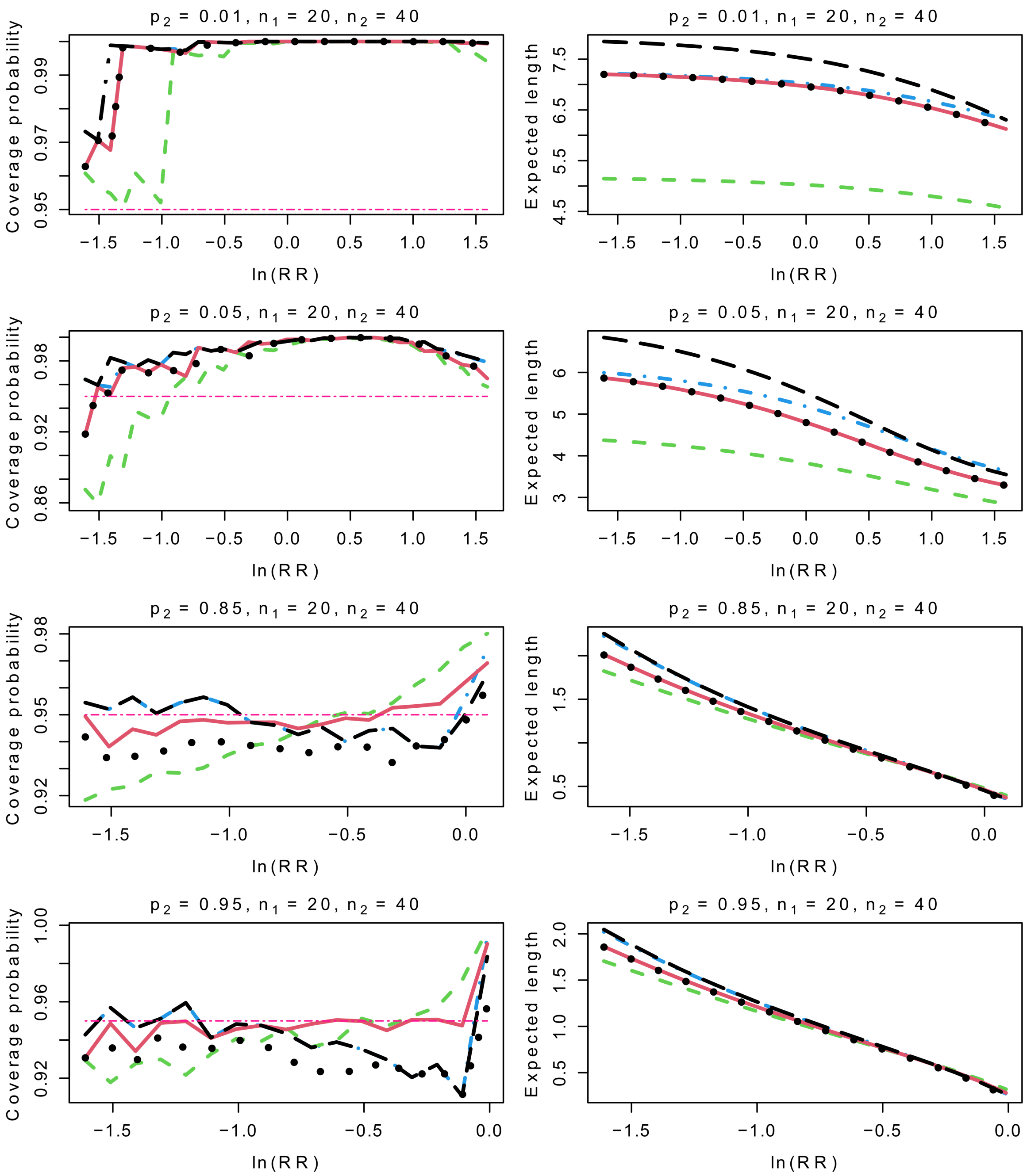

Additional Fig. 4: Comparison of the five CIs of RR with $p_2 = 0.05$, 0.15, 0.85 or 0.95, and $n_1 = 20$, $n_2 = 40$. The dot-dashed lines represent the simulation results of the Haldane interval, the long dashed lines represent the simulation results of the TACC interval, the short dashed lines represent the simulation results of the Carter interval, the dotted lines represent the simulation results of the Pettigrew interval, and the solid lines represent the simulation results of the hybrid interval. CI: Confidence interval, RR: Relative risk, TACC: Treatment arm continuity correction

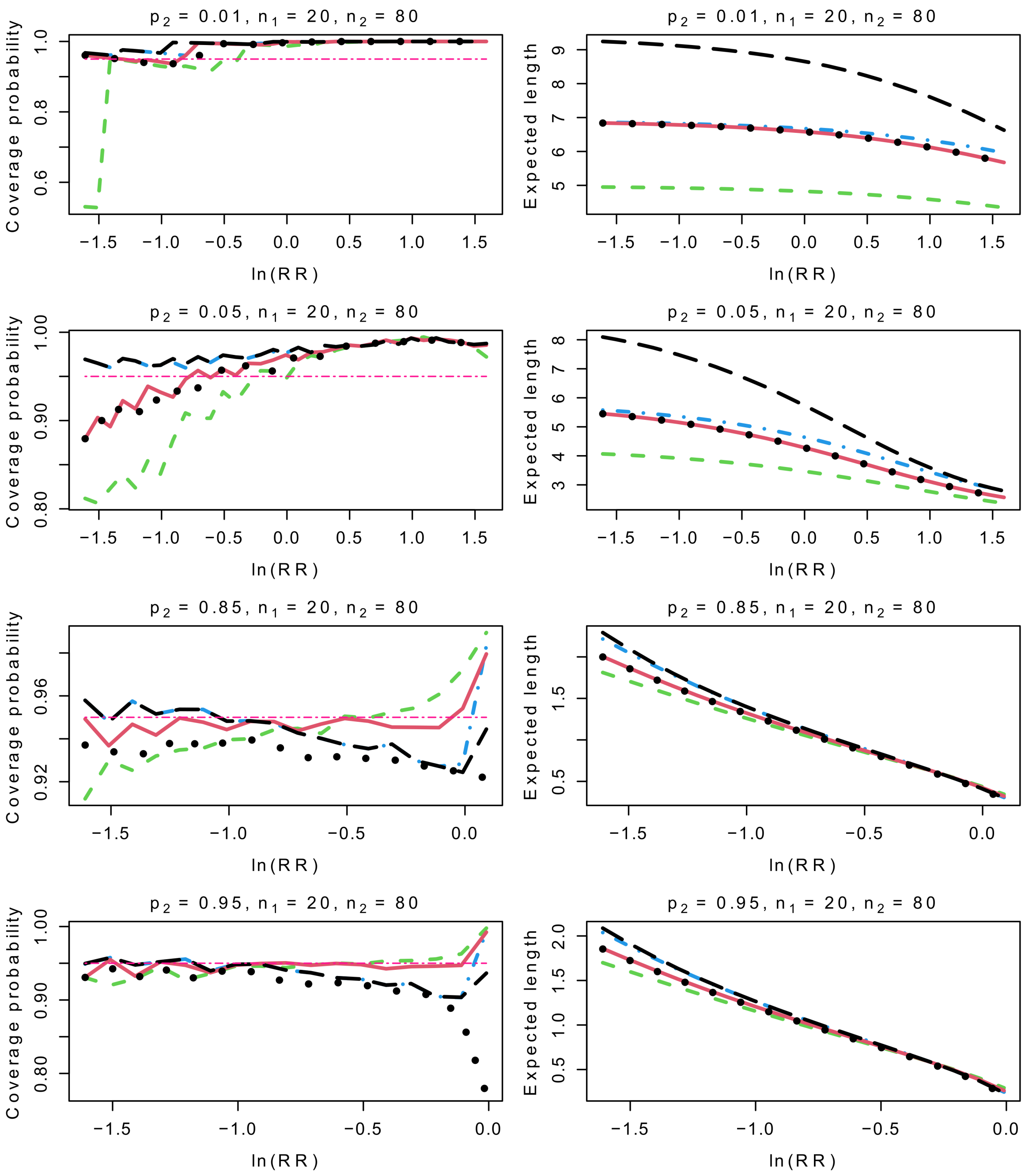

Additional Fig. 5: Comparison of the five CIs of RR with $p_2 = 0.05$, 0.15, 0.85 or 0.95, and $n_1 = 20$, $n_2 = 80$. The dot-dashed lines represent the simulation results of the Haldane interval, the long dashed lines represent the simulation results of the TACC interval, the short dashed lines represent the simulation results of the Carter interval, the dotted lines represent the simulation results of the Pettigrew interval, and the solid lines represent the simulation results of the hybrid interval. CI: Confidence interval, RR: Relative risk, TACC: Treatment arm continuity correction

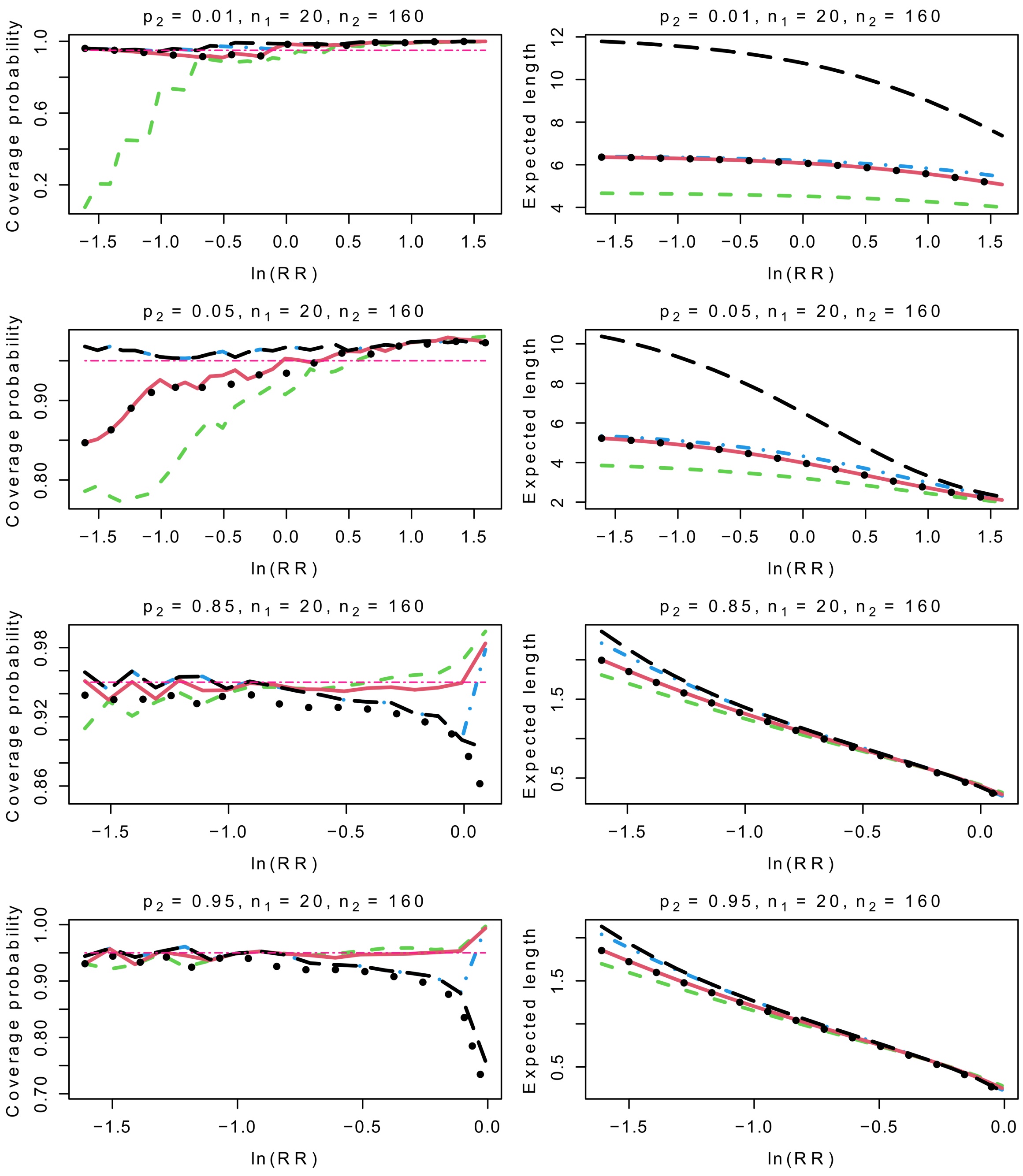

Additional Fig. 6: Comparison of the five CIs of RR with $p_2 = 0.05$, 0.15, 0.85 or 0.95, and $n_1 = 20$, $n_2 = 160$. The dot-dashed lines represent the simulation results of the Haldane interval, the long dashed lines represent the simulation results of the TACC interval, the short dashed lines represent the simulation results of the Carter interval, the dotted lines represent the simulation results of the Pettigrew interval, and the solid lines represent the simulation results of the hybrid interval. CI: Confidence interval, RR: Relative risk, TACC: Treatment arm continuity correction

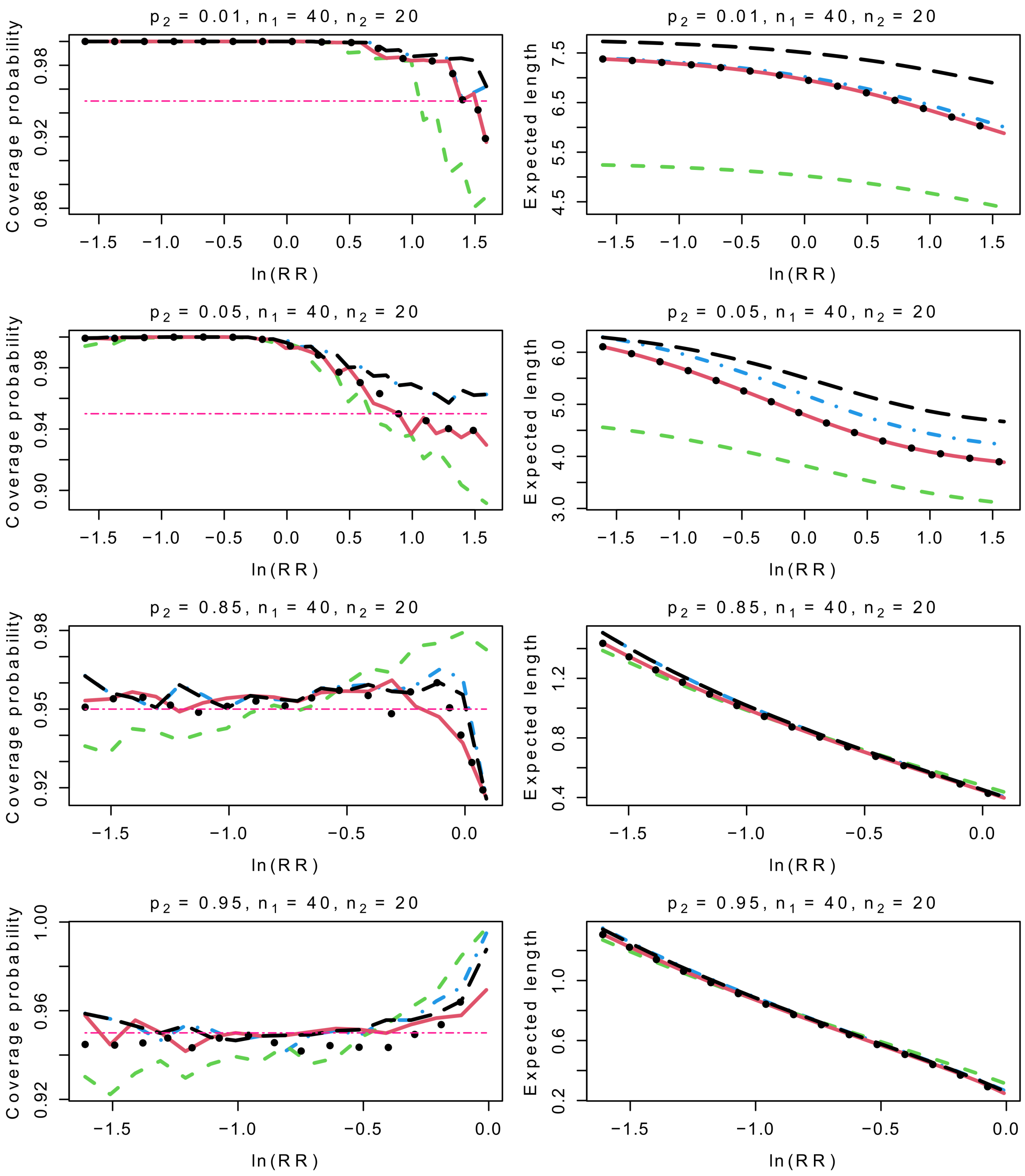

Additional Fig. 7: Comparison of the five CIs of RR with $p_2 = 0.05$, 0.15, 0.85 or 0.95, and $n_1 = 40$, $n_2 = 20$. The dot-dashed lines represent the simulation results of the Haldane interval, the long dashed lines represent the simulation results of the TACC interval, the short dashed lines represent the simulation results of the Carter interval, the dotted lines represent the simulation results of the Pettigrew interval, and the solid lines represent the simulation results of the hybrid interval. CI: Confidence interval, RR: Relative risk, TACC: Treatment arm continuity correction

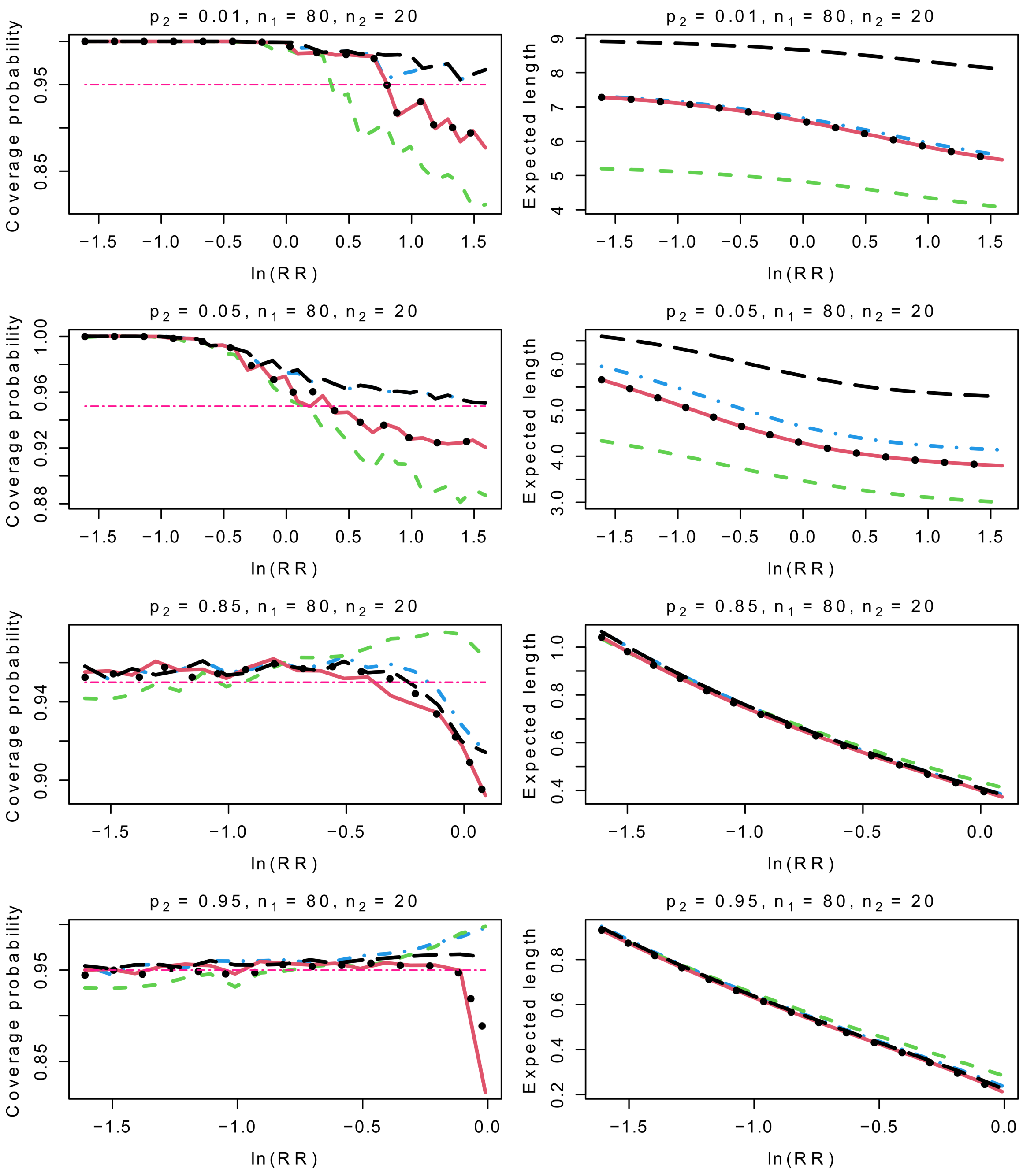

Additional Fig. 8: Comparison of the five CIs of RR with $p_2 = 0.05$, 0.15, 0.85 or 0.95, and $n_1 = 80$, $n_2 = 20$. The dot-dashed lines represent the simulation results of the Haldane interval, the long dashed lines represent the simulation results of the TACC interval, the short dashed lines represent the simulation results of the Carter interval, the dotted lines represent the simulation results of the Pettigrew interval, and the solid lines represent the simulation results of the hybrid interval. CI: Confidence interval, RR: Relative risk, TACC: Treatment arm continuity correction

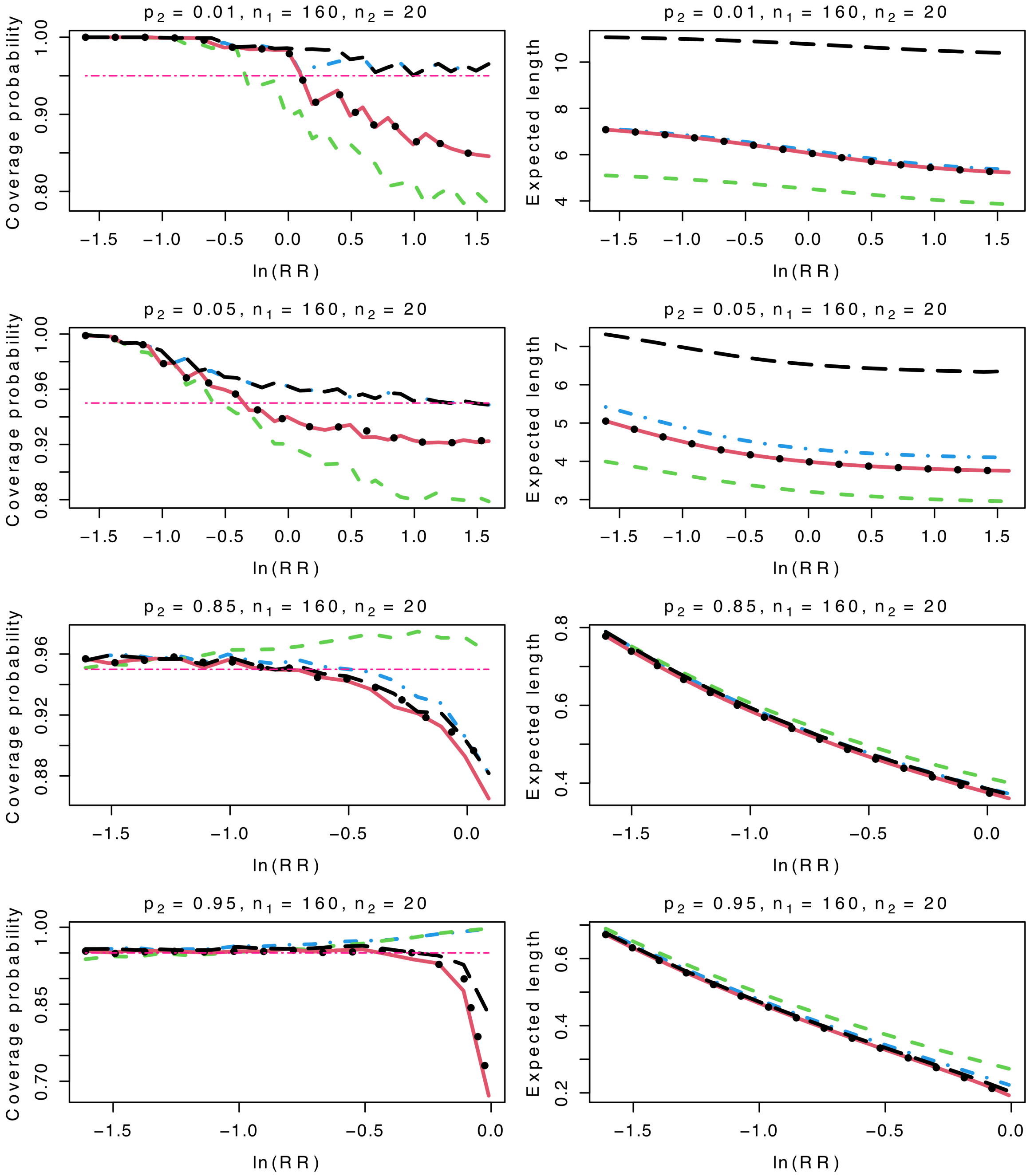

Additional Fig. 9: Comparison of the five CIs of RR with $p_2 = 0.05$, 0.15, 0.85 or 0.95, and $n_1 = 160$, $n_2 = 20$. The dot-dashed lines represent the simulation results of the Haldane interval, the long dashed lines represent the simulation results of the TACC interval, the short dashed lines represent the simulation results of the Carter interval, the dotted lines represent the simulation results of the Pettigrew interval, and the solid lines represent the simulation results of the hybrid interval. CI: Confidence interval, RR: Relative risk, TACC: Treatment arm continuity correction

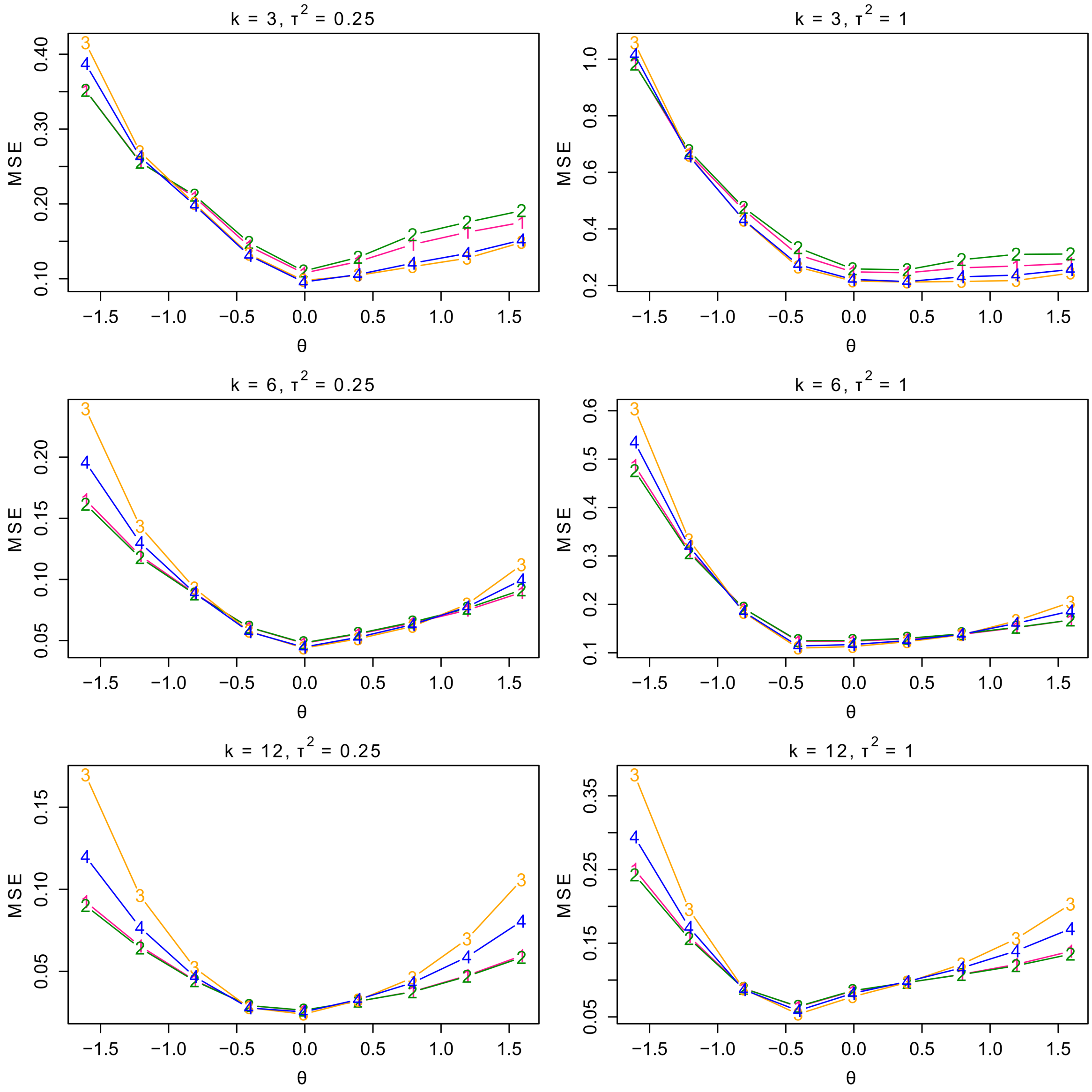

Additional Fig. 10: Comparison of the four methods with $k = 3$, 6 or 12, $\tau^2 = 0.25$ or 1. "1" represents the results of the random-effects model with the Haldane estimator, "2" represents the results of the random-effects model with the TACC estimator, "3" represents the results of the random-effects model with the Carter estimator, and "4" represents the results of the random-effects model with the Pettigrew estimator. CI: TACC: Treatment arm continuity correction, MSE: Mean squared error

that the overall effect size is 0.11, and the 95% CI is [0.02, 0.53]. Last but not least, the GLMM with the logit link provides the overall effect size of 0.23 with the 95% CI being $[0.05, 0.63]$, and the GLMM with the probit link provides the overall effect size of 0.22 with the 95% CI being $[0.06, 0.53]$.

Appendix 7: R code for analyzing COVID-19 data

```
# Effect size: RR
#  Without the double-zero-event studies
# Random-effects model with the Haldane estimator
library(meta)
Covid1 = read.table(header = TRUE, as.is = TRUE, text = "
         author       event.e  n.e  event.c  n.c
         Bai          0        76   12       42
         Burke        0        13   2        2
         Liu          0        17   2        3
         Cheng        5        47   7        36
         Heinzerling  0        4    3        33
         ")
meta1 = metabin(event.e, n.e , event.c, n.c, data = Covid1, sm = "RR",
        incr = 0.5, studlab = author, RR.Cochrane = 1, comb.fixed = F)
forest(meta1)

# Random-effects model with the TACC estimator
Covid2 = read.table(header=TRUE, as.is=TRUE, text="
         author       event.e  n.e  event.c  n.c
         Bai          0        76   12       42
         Burke        0        13   2        2
         Liu          0        17   2        3
```

Study	Treatment Event	Treatment Total	Control Event	Control Total	OR	[95% CI]	Weight
1	0	76	12	42	0.02	[0.00, 0.28]	20.2%
2	0	13	2	2	0.01	[0.00, 0.47]	14.0%
3	0	17	2	3	0.02	[0.00, 0.55]	17.0%
4	5	47	7	36	0.49	[0.14, 1.71]	30.0%
5	0	4	3	33	0.97	[0.04, 22.02]	18.8%
6	0	50	0	76			0.0%
7	0	41	0	37			0.0%
Overall					0.09	[0.01, 0.67]	100.0%

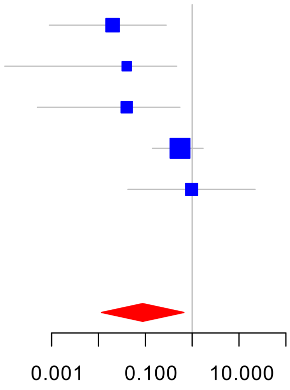

Study	Treatment Event	Treatment Total	Control Event	Control Total	OR	[95% CI]	Weight
1	0	76	12	42	0.02	[0.00, 0.26]	25.2%
2	0	13	2	2	0.00	[0.00, 1.49]	9.1%
3	0	17	2	3	0.03	[0.00, 0.59]	20.9%
4	5	47	7	36	0.49	[0.14, 1.71]	36.1%
5	0	4	3	33	0.21	[0.00, 95.99]	8.7%
6	0	50	0	76			0.0%
7	0	41	0	37			0.0%
Overall					0.07	[0.01, 0.54]	100.0%

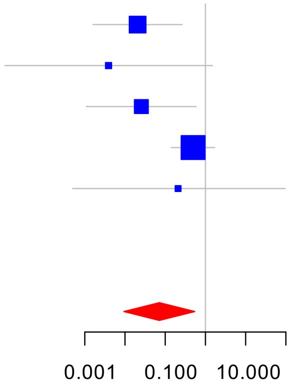

Study	Treatment Event	Treatment Total	Control Event	Control Total	OR	[95% CI]	Weight
1	0	76	12	42	0.02	[0.00, 0.28]	23.3%
2	0	13	2	2	0.01	[0.00, 0.73]	13.7%
3	0	17	2	3	0.02	[0.00, 0.56]	21.7%
4	5	47	7	36	0.49	[0.14, 1.71]	35.4%
5	0	4	3	33	0.11	[0.00, 473.39]	5.9%
6	0	50	0	76			0.0%
7	0	41	0	37			0.0%
Overall					0.06	[0.01, 0.51]	100.0%

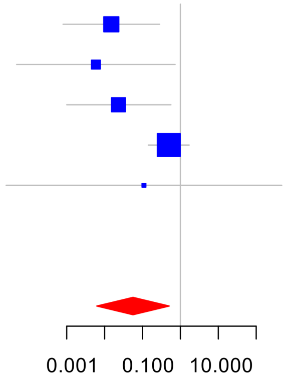

Additional Fig. 11: Meta-analyses of COVID-19 data without the double-zero-event studies by applying Haldane's continuity correction (top), the treatment arm continuity correction (middle), and the empirical continuity correction (bottom). COVID-19: Coronavirus disease 2019, OR: Odds ratio, CI: Confidence interval

Study	Treatment Event	Treatment Total	Control Event	Control Total	OR	[95% CI]	Weight
1	0	76	12	42	0.02	[0.00, 0.28]	15.6%
2	0	13	2	2	0.01	[0.00, 0.47]	10.1%
3	0	17	2	3	0.02	[0.00, 0.55]	12.7%
4	5	47	7	36	0.49	[0.14, 1.71]	25.8%
5	0	4	3	33	0.97	[0.04, 22.02]	14.2%
6	0	50	0	76	1.51	[0.03, 77.58]	10.8%
7	0	41	0	37	0.90	[0.02, 46.68]	10.8%
Overall					0.16	[0.03, 0.82]	100.0%

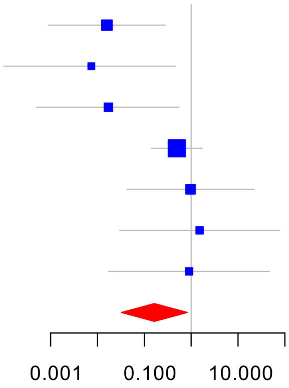

Study	Treatment Event	Treatment Total	Control Event	Control Total	OR	[95% CI]	Weight
1	0	76	12	42	0.02	[0.00, 0.26]	19.4%
2	0	13	2	2	0.00	[0.00, 1.49]	5.9%
3	0	17	2	3	0.03	[0.00, 0.59]	15.3%
4	5	47	7	36	0.49	[0.14, 1.71]	31.6%
5	0	4	3	33	0.21	[0.00, 95.99]	5.6%
6	0	50	0	76	1.00	[0.02, 55.81]	11.0%
7	0	41	0	37	1.00	[0.02, 51.93]	11.3%
Overall					0.14	[0.03, 0.68]	100.0%

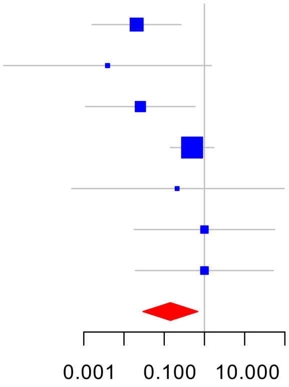

Study	Treatment Event	Treatment Total	Control Event	Control Total	OR	[95% CI]	Weight
1	0	76	12	42	0.02	[0.00, 0.28]	17.5%
2	0	13	2	2	0.01	[0.00, 0.73]	8.9%
3	0	17	2	3	0.02	[0.00, 0.56]	15.9%
4	5	47	7	36	0.49	[0.14, 1.71]	33.4%
5	0	4	3	33	0.11	[0.00, 473.39]	3.4%
6	0	50	0	76	0.50	[0.01, 47.91]	9.6%
7	0	41	0	37	0.50	[0.01, 30.75]	11.2%
Overall					0.11	[0.02, 0.53]	100.0%

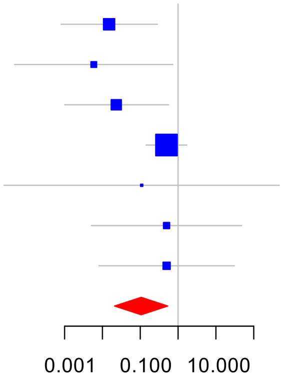

Additional Fig. 12: Meta-analyses of COVID-19 data with the double-zero-event studies by applying Haldane's continuity correction (top), the treatment arm continuity correction (middle), and the empirical continuity correction (bottom). COVID-19: Coronavirus disease 2019, OR: Odds ratio, CI: Confidence interval

```
         Cheng        5        47   7        36
         Heinzerling  0        4    3        33
         ")

meta2 = metabin(event.e ,n.e, event.c, n.c, data=Covid2, sm = "RR",
        incr = "TACC", studlab = author,
        RR.Cochrane = 1, comb.fixed = F)
forest(meta2)


# Random-effects model with the hybrid estimator
Covid3 = read.table(header = TRUE, as.is = TRUE, text = "
         author       event.e  n.e  event.c  n.c
         Bai          0.5      77   12.5     42.5
         Burke        0.5      14   2.5      2.5
         Liu          0.5      18   2.5      3.5
         Cheng        5.5      48   7.5      36.5
         Heinzerling  0.5      5    3.5      33.5
         ")
meta3 = metabin(event.e, n.e, event.c, n.c, data = Covid3, sm = "RR",
        studlab = author, incr = 0, comb.fixed = F)
forest(meta3)

# The GLMM
library(altmeta)
sid = c(1,1,2,2,3,3,4,4,5,5)      # study id
tid = c(0,1,0,1,0,1,0,1,0,1)      # 0/1: control/experiment group
e = c(12,0,2,0,2,0,7,5,3,0)       # the number of events
n = c(42,76,2,13,3,17,36,47,33,4) # the number of samples
```

```
data_glmm = data.frame(sid, tid, e, n)
glmm_logit = meta.biv(sid, tid, e, n, data_glmm, link = "logit",
             alpha = 0.05, b.iter = 1000)
glmm_probit = meta.biv(sid, tid, e, n, data_glmm, link = "probit",
              alpha = 0.05, b.iter = 1000)
```

```
# With the double-zero-event studies
# Random-effects model with the Haldane estimator
library(meta)
Covid1 = read.table(header = TRUE, as.is = TRUE, text = "
         author      event.e  n.e  event.c  n.c
         Bai         0        76   12       42
         Burke       0        13   2        2
         Liu         0        17   2        3
         Cheng       5        47   7        36
         Heinzerling 0        4    3        33
         Burke       0        50   0        76
         Burke       0        41   0        37
         ")
meta1 = metabin(event.e, n.e, event.c, n.c, data = Covid1,
        sm="RR",incr = 0.5, allstudies = T, studlab = author,
        RR.Cochrane = 1, comb.fixed = F)
forest(meta1)

# Random-effects model with the TACC estimator
Covid2 = read.table(header=TRUE, as.is=TRUE, text="
```

```
          author       event.e  n.e  event.c  n.c
          Bai          0        76   12       42
          Burke        0        13   2        2
          Liu          0        17   2        3
          Cheng        5        47   7        36
          Heinzerling  0        4    3        33
          Burke        0        50   0        76
          Burke        0        41   0        37
          ")
meta2 = metabin(event.e, n.e, event.c, n.c, data = Covid2,
                sm="RR",incr = "TACC", allstudies = T, studlab = author,
                RR.Cochrane = 1, comb.fixed = F)
forest(meta2)

# Random-effects model with the hybrid estimator
Covid3 = read.table(header = TRUE, as.is = TRUE, text = "
          author       event.e  n.e  event.c  n.c
          Bai          0.5      77   12.5     42.5
          Burke        0.5      14   2.5      2.5
          Liu          0.5      18   2.5      3.5
          Cheng        5.5      48   7.5      36.5
          Heinzerling  0.5      5    3.5      33.5
          Burke        0.5      51   0.5      76.5
          Burke        0.5      42   0.5      37.5
          ")
meta3 = metabin(event.e ,n.e, event.c, n.c, data = Covid3,
        sm = "RR", studlab = author, incr = 0, comb.fixed = F)
forest(meta3)
```

```
# The GLMM
# study id
sid = c(1,1,2,2,3,3,4,4,5,5,6,6,7,7)
# 0/1: control/experiment group
tid = c(0,1,0,1,0,1,0,1,0,1,0,1,0,1)
# the number of events
e = c(12,0,2,0,2,0,7,5,3,0,0,0,0,0)
# the number of samples
n = c(42,76,2,13,3,17,36,47,33,4,76,50,37,41)
data_glmm = data.frame(sid, tid, e, n)
glmm_logit = meta.biv(sid, tid, e, n, data_glmm, link = "logit",
             alpha = 0.05, b.iter = 1000)
glmm_probit = meta.biv(sid, tid, e, n, data_glmm, link = "probit",
              alpha = 0.05, b.iter = 1000)
```

```
# Effect size: OR
#  Without the double-zero-event studies
# Random-effects model with Haldane's continuity correction
library(meta)
Covid1 <- read.table(header=TRUE, as.is=TRUE, text="
         author       event.e  n.e  event.c  n.c
         Bai          0        76   12       42
         Burke        0        13   2        2
         Liu          0        17   2        3
         Cheng        5        47   7        36
         Heinzerling  0        4    3        33
         ")
```

```
meta1<- metabin(event.e ,n.e, event.c, n.c, data=Covid1,
                sm = "OR", incr = 0.5, studlab = author,
                comb.fixed = F)
forest(meta1)

# Random-effects model with the treatment arm continuity correction
Covid2 = read.table(header=TRUE, as.is=TRUE, text="
         author       event.e  n.e  event.c  n.c
         Bai          0        76   12       42
         Burke        0        13   2        2
         Liu          0        17   2        3
         Cheng        5        47   7        36
         Heinzerling  0        4    3        33
         ")

meta2 = metabin(event.e ,n.e, event.c, n.c, data=Covid2, sm = "OR",
                incr = "TACC", studlab = author,
                comb.fixed = F)
forest(meta2)

# Random-effects model with the empirical continuity correction
Covid3 = read.table(header=TRUE, as.is=TRUE, text="
         author       event.e  n.e  event.c  n.c
         Bai          0        76   12       42
         Burke        0        13   2        2
         Liu          0        17   2        3
         Cheng        5        47   7        36
         Heinzerling  0        4    3        33
```

```
         ")
Omega = (5*29)/(42*7)
for(i in 1:nrow(Covid3))
{
  group.ratio = Covid3[i,5]/Covid3[i,3]
  if(Covid3[i,2]==0|Covid3[i,4]==0)
  {
    Covid3[i,2] = Covid3[i,2] + Omega/(group.ratio + Omega)
    Covid3[i,3] = Covid3[i,3] + 2*Omega/(group.ratio + Omega)
    Covid3[i,4] = Covid3[i,4] + group.ratio/(group.ratio + Omega)
    Covid3[i,5] = Covid3[i,5] + 2*group.ratio/(group.ratio + Omega)
  }
}
meta3 = metabin(event.e ,n.e, event.c, n.c, data=Covid3, sm = "OR",
                incr = 0, studlab = author,
                comb.fixed = F)
forest(meta3)

# The GLMM
library(altmeta)
sid = c(1,1,2,2,3,3,4,4,5,5)      # study id
tid = c(0,1,0,1,0,1,0,1,0,1)      # 0/1: control/experiment group
e = c(12,0,2,0,2,0,7,5,3,0)       # the number of events
n = c(42,76,2,13,3,17,36,47,33,4) # the number of samples
data_glmm = data.frame(sid, tid, e, n)
glmm_logit = meta.biv(sid, tid, e, n, data_glmm, link = "logit",
             alpha = 0.05, b.iter = 1000)
glmm_probit = meta.biv(sid, tid, e, n, data_glmm, link = "probit",
              alpha = 0.05, b.iter = 1000)
```

```
# Effect size: OR
#  With the double-zero-event studies
# Random-effects model with Haldane's continuity correction
Covid1 = read.table(header=TRUE, as.is=TRUE, text="
         author       event.e  n.e  event.c  n.c
         Bai          0        76   12       42
         Burke        0        13   2        2
         Liu          0        17   2        3
         Cheng        5        47   7        36
         Heinzerling  0        4    3        33
         Burke        0        50   0        76
         Burke        0        41   0        37
         ")
meta1 = metabin(event.e, n.e, event.c, n.c, data = Covid1,
                sm="OR",incr = 0.5, allstudies = T, studlab = author,
                comb.fixed = F)

# Random-effects model with the treatment arm continuity correction
Covid2 = read.table(header=TRUE, as.is=TRUE, text="
         author       event.e  n.e  event.c  n.c
         Bai          0        76   12       42
         Burke        0        13   2        2
         Liu          0        17   2        3
         Cheng        5        47   7        36
         Heinzerling  0        4    3        33
         Burke        0        50   0        76
```

```
         Burke        0        41   0        37
         ")
meta2 = metabin(event.e, n.e, event.c, n.c, data = Covid2,
                sm="OR",incr = "TACC", allstudies = T, studlab = author,
                comb.fixed = F)
forest(meta2)

# Random-effects model with the empirical continuity correction
Covid3 = read.table(header=TRUE, as.is=TRUE, text="
         author       event.e  n.e  event.c  n.c
         Bai          0        76   12       42
         Burke        0        13   2        2
         Liu          0        17   2        3
         Cheng        5        47   7        36
         Heinzerling  0        4    3        33
         Burke        0        50   0        76
         Burke        0        41   0        37
         ")

Omega = (5*29)/(42*7)
for(i in 1:nrow(Covid3))
{
  group.ratio = Covid3[i,5]/Covid3[i,3]
  if(Covid3[i,2]==0|Covid3[i,4]==0)
  {
    Covid3[i,2] = Covid3[i,2] + Omega/(group.ratio + Omega)
    Covid3[i,3] = Covid3[i,3] + 2*Omega/(group.ratio + Omega)
    Covid3[i,4] = Covid3[i,4] + group.ratio/(group.ratio + Omega)
    Covid3[i,5] = Covid3[i,5] + 2*group.ratio/(group.ratio + Omega)
```

```
  }
}

meta3 <- metabin(event.e ,n.e, event.c, n.c, data = Covid3, allstudies = T,
                 sm = "OR", studlab = author, incr = 0, comb.fixed = F)
forest(meta3)

# The GLMM
# study id
sid = c(1,1,2,2,3,3,4,4,5,5,6,6,7,7)
# 0/1: control/experiment group
tid = c(0,1,0,1,0,1,0,1,0,1,0,1,0,1)
# the number of events
e = c(12,0,2,0,2,0,7,5,3,0,0,0,0,0)
# the number of samples
n = c(42,76,2,13,3,17,36,47,33,4,76,50,37,41)
data_glmm = data.frame(sid, tid, e, n)
glmm_logit = meta.biv(sid, tid, e, n, data_glmm, link = "logit",
             alpha = 0.05, b.iter = 1000)
glmm_probit = meta.biv(sid, tid, e, n, data_glmm, link = "probit",
              alpha = 0.05, b.iter = 1000)
```
